# Finite element analysis of warpage and debonding behavior in RDL-first and molding-first fan-out panel-level packaging

**DOI:** 10.1038/s41598-026-47275-2

**Published:** 2026-06-15

**Authors:** Chih-Ping Hu, Ming-Hsien Shih, Chun-Chieh Hung, Sheng-Jye Hwang

**Affiliations:** 1 Department of Mechanical Engineering, National Cheng Kung University, Tainan, Taiwan; 2 Department of Mechanical Engineering, Southern Taiwan University of Science and Technology, Tainan, 710301 Taiwan

**Keywords:** Fan-out panel-level packaging, Process modeling technology, Warpage, Debonding, Molding-first, RDL-first, Engineering, Materials science

## Abstract

This study investigates the applicability of various carrier materials in fan-out panel-level packaging (FOPLP) processes through finite element analysis (FEA). Numerical simulations were performed for RDL-first and molding-first process flows to evaluate warpage and stress distribution across different carrier types, including steel, glass, and ceramic. Two panel dimensions, 600 $\:\times\:$ 700 mm and 680 $\:\times\:$ 680 mm, were modeled under varying manufacturing processes, temperature settings, and material properties. The simulations incorporated both mechanical and chemical shrinkage effects, with the molding-first process modeled from compression molding to the debonding stage. The element birth and death technique was implemented to account for material addition and removal during processing, thereby enhancing simulation accuracy. The results indicate that the average reference temperature provides the lowest prediction error in the RDL-first process, while maximum von Mises stress consistently occurs in the RM 1 and WAL layers. Furthermore, a significant increase in warpage is observed during the debonding stage in the molding-first process. A comparative analysis between simulation and experimental results demonstrates a high level of agreement, confirming the validity and reliability of the modeling approach. By systematically examining the thermo-mechanical behavior of multiple carrier materials in different process flows, this work establishes a comprehensive design guideline for carrier selection and process optimization in advanced FOPLP manufacturing.

## Introduction

Warpage and residual stress have become critical challenges in modern integrated circuit (IC) packaging as device architectures continue to evolve toward thinner profiles and higher integration density. To meet the demand for compact and high-performance systems, advanced packaging technologies increasingly rely on vertical stacking and multilayer interconnect structures. However, the heterogeneous nature of packaging materials introduces significant mismatches in mechanical and thermal properties, which can lead to deformation, stress accumulation, and reduced manufacturing yield.

To ensure structural integrity and enable reliable electrical interconnection, semiconductor devices are typically encapsulated using polymeric or ceramic materials that provide both mechanical protection and signal transmission pathways. Among various advanced packaging solutions, fan-out panel-level packaging (FOPLP) has gained considerable attention due to its capability to achieve high input/output (I/O) density while maintaining a compact form factor. These advantages make FOPLP particularly attractive for emerging applications, including power electronics, 5G communication modules, and low-earth orbit satellite systems.

Despite its advantages, fan-out packaging introduces significant thermo-mechanical challenges due to its multilayer structure and high-density redistribution layer (RDL) design. The coexistence of materials with different coefficients of thermal expansion (CTE) intensifies thermal mismatch, particularly under processing temperature variations. This mismatch leads to the accumulation of residual stresses, which can manifest as package warpage or even structural damage, especially in thin and highly integrated packages. As device thickness continues to decrease, the reduction in structural stiffness further amplifies deformation, making warpage control a critical issue for packaging reliability. These phenomena have been widely reported in the literature, where thermo-mechanical mismatch is identified as a dominant factor affecting structural integrity in advanced fan-out configurations^1^.

**Fig. 1:**
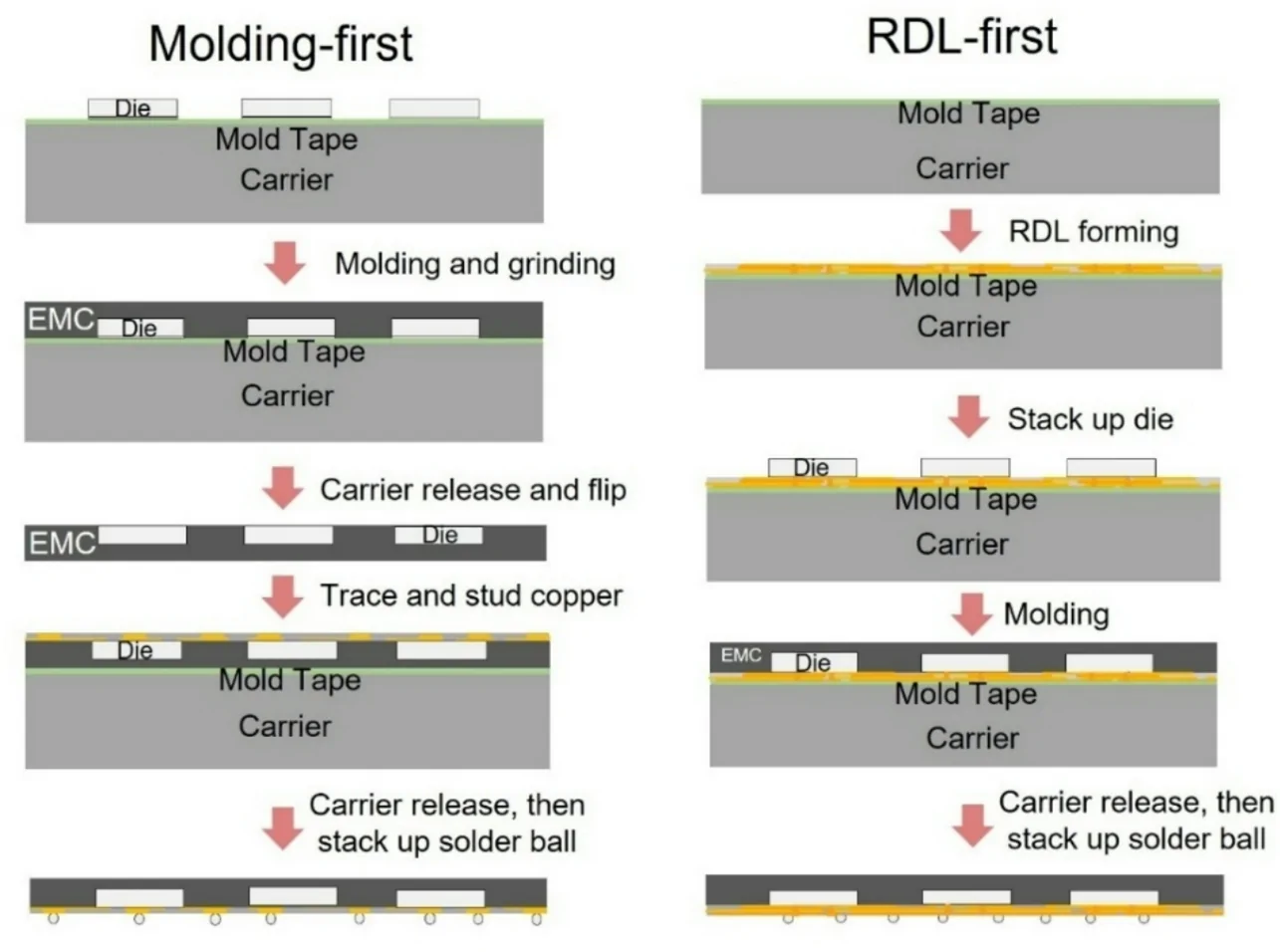
Process flowchart of molding-first and RDL-first fan-out process.

From a process perspective, fan-out packaging can generally be divided into two main approaches: molding-first and RDL-first processes, as illustrated in Fig. 1. Each process introduces distinct thermo-mechanical characteristics due to differences in material addition sequence and thermal history. In this study, particular attention is given to warpage induced by RDL formation in the RDL-first process, as well as deformation associated with molding and subsequent debonding in the molding-first process. Considering the strong coupling between material behavior, thermal loading, and process sequence, a comprehensive review of the underlying theoretical models and prior research is necessary to establish a solid foundation for accurate warpage prediction.

The theoretical foundation of warpage and thin-film stress analysis dates back to 1909, when Stoney introduced an equation for estimating the internal stress of thin films based on substrate curvature, known as the Stoney equation^2^. In 1925, Timoshenko developed a thermal stress theory for bilayer heterogeneous structures, providing analytical models for curvature and stress distribution^3^. Building on these early studies, Suhir applied classical plate theory to derive analytical solutions for multilayer packaging warpage under thermal loading, emphasizing the combined effects of material thermal expansion properties and structural geometry^4^. Schapery later formulated a theoretical framework for calculating the effective coefficient of thermal expansion (CTE) in heterogeneous composites, forming a fundamental basis for predicting residual stress and deformation^5^.

Regarding structural factors in fan-out packaging, Che and Chen identified the copper content within redistribution layers (RDL) as a critical determinant of warpage^6,7^. Higher copper volume fractions exacerbate thermal mismatch-induced stress concentration, increasing package deformation. Through finite element analysis and experimental validation, the study confirmed the sensitivity of warpage to copper ratio. It proposed practical guidelines for material configuration and geometric optimization, contributing to a more robust understanding and control of warpage in fan-out wafer-level packaging (FOWLP).

Beyond material composition and geometry, the temporal sequence of processing steps and the addition or removal of materials significantly influence warpage evolution. Wu employed finite element analysis with the element birth and death technique to dynamically simulate key process stages, including carrier removal, grinding, and annealing^8^. This approach effectively captured the impact of material changes on warpage, with simulation results exhibiting less than 10% deviation from experimental data. These findings underscore the importance of accurately reproducing process chronology in numerical modeling to enhance predictive reliability.

Equivalent material modeling often reduces simulation complexity while preserving representative thermo-mechanical behavior. However, its accuracy depends heavily on properly defining the process reference temperature. Focusing on RDL-first structures, Lee emphasized that when a complex RDL stack is simplified into a homogeneous equivalent layer, the reference temperature must reflect the actual thermal history of each constituent material^9^. Failure to incorporate appropriate temperature baselines during material addition or removal results in an inaccurate representation of CTE mismatch and compromised warpage prediction.

In addition, Liang conducted a systematic comparison to demonstrate that simplified reference temperature assumptions fail to capture the cumulative stress evolution in multilayer RDL stacking^10^. Using room temperature or the dielectric curing temperature as a constant reference may introduce substantial errors in thermal stress estimation, leading to discrepancies between simulation and experimental results. Even when combining equivalent material properties with reference temperature settings, predicted maximum warpage remained significantly lower than experimental measurements. These results highlight the necessity of dynamically adjusting the reference temperature according to the actual process sequence and thermal profile to preserve physical consistency and simulation credibility.

Carrier selection also plays a decisive role in package warpage and structural reliability. Through finite element simulations, Lee analyzed warpage at various processing stages and identified carrier properties as a key factor^11^. The study revealed that Alloy 42 exhibited lower warpage compared to other materials. Considering overall cost, oxidation resistance, and thermal conductivity, Alloy 42 and SUS304 emerged as favorable options, illustrating how material-level decisions complement process optimization strategies.

From the material modeling perspective in molding-first fan-out packaging, effective thermal expansion methods can unify thermal and chemical shrinkage into a single equivalent expansion behavior, facilitating warpage simulation across multilayer structures. Tsai applied this approach to characterize thermal behavior across layers, enabling finite element analysis to capture global deformation behavior effectively^12^. Building on this foundation, Teng integrated viscosity, reaction kinetics, and pressure–volume–temperature–cure (*P–V–T–C*) models to show that cure shrinkage contributes more significantly to warpage than thermal expansion alone^13^. Neglecting the cure effects leads to a substantial underestimation of warpage. Model validation using thin small outline package (TSOP) and low-profile quad flat package (LQFP) packages confirmed that simulation results could align closely with experimental results only by incorporating *P–V–T–C* behavior.

Focusing on the encapsulation stage, Liang and Deng employed a *P–V–T–C* model to incorporate both the cure kinetics and thermal expansion behavior of epoxy molding compound (EMC)^10,14^. EMC properties were characterized through differential scanning calorimetry (DSC) and *P–V–T–C* testing, enabling the model to reproduce post-molding warpage accurately. These results confirm that accurate warpage prediction in molding-first processes requires simultaneous consideration of cure-induced shrinkage and thermal mismatch.

In the debonding process, nonlinear warpage may be amplified by variation in debonding paths and geometric uncertainties. Chen utilized the element birth and death method in ANSYS to simulate different debonding scenarios and identify their impact on warpage^15^. In parallel, Lo and Lee investigated warpage in very fine-pitch BGA (VFBGA) and system-in-package (SiP) structures, respectively, using Moldex3D with advanced viscoelastic and thermo-mechanical material models^16,17^. Their results demonstrated a strong correlation between simulations and experimental data, reaffirming that robust numerical tools must be integrated with realistic material models to ensure predictive accuracy.

To further validate the robustness of numerical methods in polymer processing, it is instructive to consider the synergy between simulation and experiment in related additive manufacturing fields. Khosravani et al. investigated components fabricated via the stereolithography (SLA) technique using UV sensitive resin material, employing FEA to accurately predict stress distributions and fracture loads across various notch geometries^18^. Regarding functional performance, it was demonstrated that perforation ratios, porous material layers, and the air gap significantly influence the sound absorption coefficient of multilayered structures^19^. For interfacial strength, designing the overlap area of single-lap joints into a stepped-shape configuration effectively enhanced the load-bearing capacity^20^. Additionally, Lyu et al. highlighted that post-processing significantly improves interfacial shear strength^21^, providing critical insights for optimizing the bonding of heterogeneous interfaces in packaging processes.

### Research gap

Due to the consistent underestimation of experimentally measured warpage when using conventional reference temperature settings in previous RDL-first studies, and the lack of systematic investigation into material-dependent effects and optimal carrier selection, this study aims to systematically investigate the warpage behavior in fan-out panel-level packaging (FOPLP) under both RDL-first and molding-first process flows. This study introduces a modeling strategy based on an average reference temperature, improving the physical consistency and reproducibility of warpage prediction in RDL-first packaging. A unified simulation framework is developed to capture critical warpage mechanisms in both RDL-first and molding-first processes, including RDL formation, encapsulation, and post-debonding deformation.

The proposed models incorporate experimentally measured material properties and account for both cure-induced and thermal shrinkage effects. Furthermore, a systematic comparison of carrier materials is conducted, covering steels, glass, and ceramics, in order to establish practical guidelines for material selection across different panel sizes and process conditions. The simulation workflow also integrates key process steps using the element birth and death method to maintain continuity and consistent boundary conditions. The strong agreement between simulation results and experimental measurements confirms the effectiveness of the proposed methodology in reducing prediction error and enhancing warpage control in advanced packaging applications.

## Theorem and methodology

The filling analysis was conducted to simulate the distribution of the melting front, molding temperature, velocity, and conversion within the cavity. Fluid flow phenomena are described by the continuity equation, momentum equation, and energy equation.

### Filling analysis theorem

#### Continuity equation

1 $$\frac{{\partial {\rm{\rho }}}}{{\partial {\rm{t}}}}{\rm{~ + ~}}\nabla \cdot \left( {{\rm{\rho \mathord{\buildrel{\lower3pt\hbox{$\scriptscriptstyle\rightharpoonup$}} \over V} }}} \right){\rm{~ = ~0}}$$

*ρ*: density; *t*: time; $${\rm{\mathord{\buildrel{\lower3pt\hbox{$\scriptscriptstyle\rightharpoonup$}} \over V} }}$$: velocity vector; *∇*: divergence operator.

#### Momentum equation

2 $${\rm{~}}\rho \left( {\frac{{\partial {\rm{\mathord{\buildrel{\lower3pt\hbox{$\scriptscriptstyle\rightharpoonup$}} \over V} }}}}{{\partial {\rm{t}}}}{\rm{~ + \mathord{\buildrel{\lower3pt\hbox{$\scriptscriptstyle\rightharpoonup$}} \over V} }} \cdot \nabla {\rm{\mathord{\buildrel{\lower3pt\hbox{$\scriptscriptstyle\rightharpoonup$}} \over V} }}} \right){\rm{~ = ~}}\nabla \cdot {\rm{\bar \bar \sigma total~ + }}~\rho \mathord{\buildrel{\lower3pt\hbox{$\scriptscriptstyle\rightharpoonup$}} \over g}$$

3 $${\rm{\bar \bar \sigma total}} = - P\bar \bar \delta + \bar \bar \tau$$

$${\rm{\bar \bar \sigma total}}$$: total stress tensor; *P*: pressure; *P*$$\bar \bar \delta$$: hydrostatic stress; $$\bar \bar \tau$$: extra stress; $$\mathord{\buildrel{\lower3pt\hbox{$\scriptscriptstyle\rightharpoonup$}} \over g}$$: gravity.

#### Energy equation

4 $${\rm{~\rho }}{{\rm{C}}_{\rm{p}}}\left( {\frac{{\partial {\rm{T}}}}{{\partial {\rm{t}}}}{\rm{ + \mathord{\buildrel{\lower3pt\hbox{$\scriptscriptstyle\rightharpoonup$}} \over V} }} \cdot \nabla {\rm{T}}} \right){\rm{~ = ~}}\nabla \cdot {\rm{k}}\nabla {\rm{T)~ + ~(}}\bar \bar \tau {\rm{:}}\nabla {\rm{\mathord{\buildrel{\lower3pt\hbox{$\scriptscriptstyle\rightharpoonup$}} \over V} )~ + ~\dot C}}\Delta {\rm{H}}$$

$\:\mathrm{C}\mathrm{p}$: specific heat of EMC; *T*: temperature; * k*: heat conductivity of EMC; $\:\dot{\mathrm{C}}$: cure rate of EMC; $\:\varDelta\:\mathrm{H}$: enthalpy change.

### Warpage analysis theorem

For polymer-based materials, the overall strain can be divided into three fundamental components: cure strain, thermal strain, and elastic strain, which can be represented as follows:

5 $$\left\{ {{\varepsilon _{Total}}} \right\}{\rm{ }} = {\rm{ }}\left\{ {{\varepsilon _{cure}}} \right\}{\rm{ }} + {\rm{ }}\left\{ {{\varepsilon _{th}}} \right\}{\rm{ }} + {\rm{ }}\left\{ {{\varepsilon _{el}}} \right\}$$

{*ε*_*Total*_}: total strain matrix; {*ε*_*cure*_}: cure-induced strain matrix; {*ε*_*th*_}: thermally induced strain matrix; {*ε*_*el*_}: elastic strain matrix.

#### Cure-induced strain

During curing, the polymer experiences a gradual reduction in specific volume. Assuming that the epoxy molding compound (EMC) initially has a unit volume and behaves as a homogeneous and isotropic material, the cure-induced strain can be determined based on the variation in specific volume, as illustrated in Fig. 3^14^.

Let the initial specific volume be represented by $\:\bar V\mathrm{0}$=1, and the final specific volume after complete cure by $\:\bar V\mathrm{C}$. The corresponding change in specific volume is then expressed as $\:\varDelta\:\bar V$:

6 $$\Delta \bar V\: = \:{\bar V_{\rm{C}}} - {\bar V_{\rm{0}}}\: = \:{\bar V_{\rm{C}}} - 1$$

7 $${\varepsilon _c}={\text{ }} - \frac{{{L_C} - {L_0}}}{{{L_0}}}={\text{ }} - \Delta {L_C}$$

8 $$~{\bar {V}_C}={\left( {1\,+\,{\varepsilon _c}} \right)^3}$$

9 $${\varepsilon _c}=~\sqrt[3]{{\Delta \bar {V}+1}} - {\text{ }}1$$

10 $$\:\left\{{\epsilon\:}_{cure}\right\}\:=\:\left[\begin{array}{ccc}\varepsilon_\mathrm{c}&\:\mathrm{0}&\:\mathrm{0}\\\:\mathrm{0}&\:\varepsilon_\mathrm{c}&\:\mathrm{0}\\\:\mathrm{0}&\:\mathrm{0}&\:\varepsilon_\mathrm{c}\end{array}\right]$$

*ε*_*c*_: cure-induced strain in one dimension; $\:L_\mathrm{0}$: initial length, the unit length is 1; $\:L_\mathrm{C}$: one-dimensional length after cured.

#### Thermal-induced strain

Thermal strain is primarily governed by the material’s coefficient of linear thermal expansion and the temperature difference. It can be expressed as:

11 $$\left\{ {{\varepsilon _{th}}} \right\}{\text{ }}={\text{ }}\left\{ \alpha \right\}~\Delta T,\Delta T=T - {T_f}$$

{*ε*_*th*_}: thermal induced matrix; $\:\left\{\alpha\:\right\}$: coefficients of linear thermal expansion matrix; $\:\varDelta\:\mathrm{T}$: temperature difference; * T*_*f*_: reference temperature, which refers to the temperature at which thermal strain is zero.

#### Elastic strain

The polymer materials are modeled as linear-elastic, homogeneous, and isotropic materials. The correlation between elastic strain and stress can be formulated by the generalized Hooke’s law, expressed as follows:

12 $$\left\{ {{\varepsilon _{el}}} \right\}{\text{ }}={\text{ }}{[S]^{ - 1}}\{ \sigma \}$$

{*σ*} = {*σ*_*xx*_ *σ*_*yy*_ *σ*_*zz*_ *σ*_*xy*_ *σ*_*yz*_ *σ*_*xz*_}^*T*^: stress matrix; [* S*]^−1^: stiffness matrix; {*ε*_*el*_}: elastic strain matrix.

### EMC material models

#### Viscosity model

**Fig. 2:**
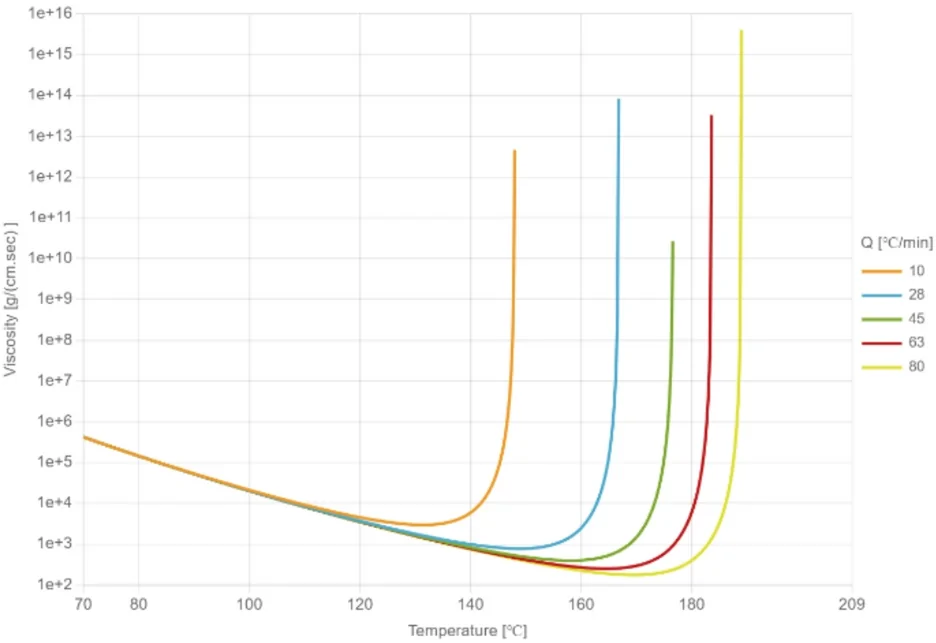
The relationship between viscosity and temperature under different heating rate.

Cross Castro-Macosko model^22^ is used to describe the viscosity behavior of EMC. The relationship between viscosity and temperature is graphically illustrated in Fig. 2, demonstrating the variation in viscosity with different temperatures.

13 $$\:\text{}\eta\text{}\mathrm{=\:}\frac{{\eta}_{\mathrm{0}}{\left(\frac{\mathrm{C}_\mathrm{g}}{\mathrm{C}_\mathrm{g}-\mathrm{C}}\right)}^{\mathrm{K}_\mathrm{1}\mathrm{\:+\:}\mathrm{K}_\mathrm{2}\mathrm{C}}}{\mathrm{1}\mathrm{+}{\left(\frac{{\eta}_{\mathrm{0}}\dot{\gamma}}{{\tau}^{\mathrm{*}}}\right)}^{\mathrm{1}-\mathrm{n}}}$$

14 $$\:{\eta}_{\mathrm{0}}\mathrm{\:=\:}\mathrm{A} \cdot \mathrm{exp}\mathrm{(}\frac{\mathrm{T}_\mathrm{b}}{\mathrm{T}}\mathrm{)},\:\mathrm{T}_\mathrm{b}\mathrm{\:=\:}\frac{\mathrm{E}_\eta}{\mathrm{R}}$$

$\:\eta$: viscosity; $\:{\eta}_{\mathrm{0}}$: zero-shear-rate viscosity; $\:\mathrm{C}_\mathrm{g}$: degree of cure at gel point; $\:\mathrm{C}$: degree of cure; $\:\dot{\gamma}$: shear strain rate; $\:{\tau\:}^{\mathrm{*}}$: critical shear stress; $\:\mathrm{n}$: power law index; *K*_1_, *K*_2_, *A* and $\:\mathrm{T}_\mathrm{b}$: model parameters; $\:\mathrm{E}_\eta$: activation energy; $\:\mathrm{R}$: ideal gas constant.

#### Cure kinetics model

**Fig. 3:**
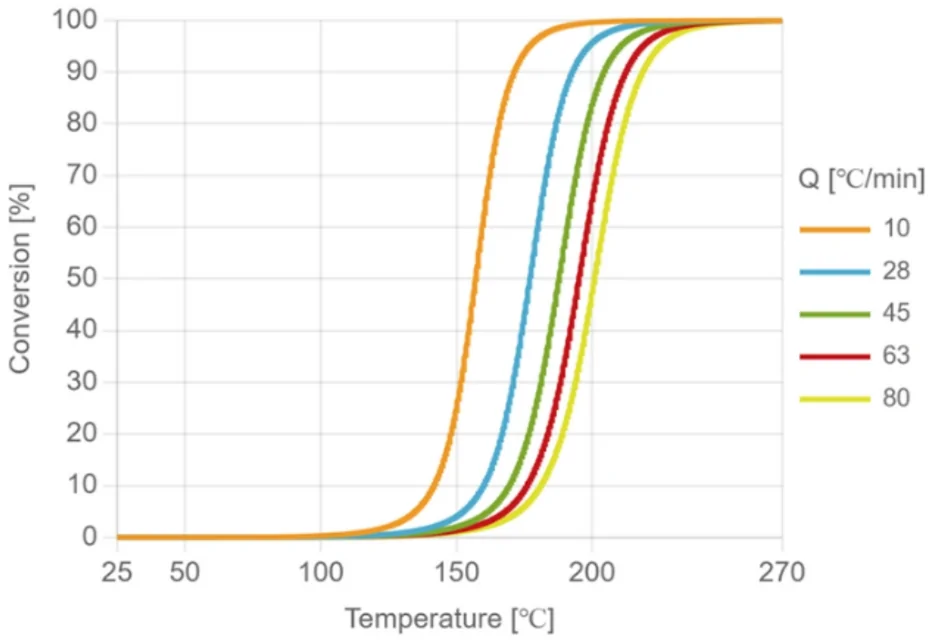
The heat absorption curve of the material as temperature increases under different rates of temperature increase.

Kamal’s model^23^ is used to describe the effect of temperature and time on the degree of cure of EMC material. The relationship between conversion rate and temperature is shown in Fig. 3.

15 $${\rm{\dot C}} = \:\frac{{{\rm{dC}}}}{{{\rm{dt}}}} = {\rm{(}}{{\rm{k}}_{\rm{1}}} + {{\rm{k}}_{\rm{2}}}{{\rm{C}}^{\rm{m}}}) \cdot {({\rm{1 - C)}}^{\rm{n}}}$$

16 $$\:\mathrm{k}_\mathrm{1}\mathrm{=\:}\mathrm{A}_\mathrm{1} \cdot \mathrm{exp}\mathrm{(}\frac{-\mathrm{T}_\mathrm{A}}{\mathrm{T}}\mathrm{)}$$

17 $${{\rm{k}}_{\rm{2}}} = \:{{\rm{A}}_{\rm{2}}} \cdot {\rm{exp(}}\frac{{ - {{\rm{T}}_{\rm{B}}}}}{{\rm{T}}})$$

$\:\dot{\mathrm{C}}$: cure rate of EMC, $\:\mathrm{k}\mathrm{1}$, $\:\mathrm{k}\mathrm{2}$: rate constant of the cure reaction, which follows Arrhenius equation; *m*, *n*, $\:A_\mathrm{1}$, $\:\mathrm{A}_\mathrm{2}$: model constants; *T*_*A*_, *T*_*B*_: activation temperature; *T*: temperature.

#### *P–V–T–C* model

**Fig. 4:**
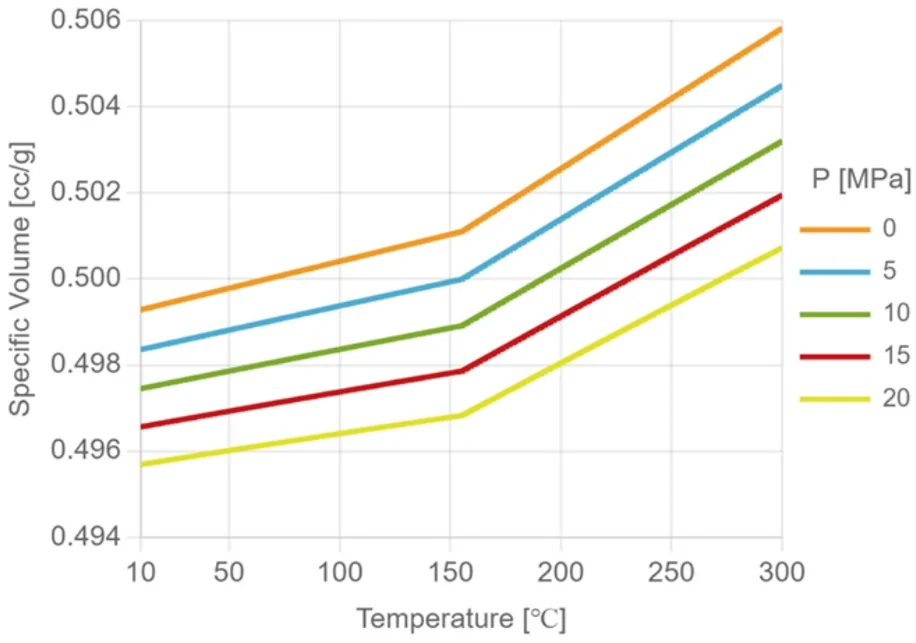
*P*–*V*–*T*–*C* relationship.

Two domain modified Tait model^20,21^ is used to describe the relation between *P*–*V*–*T*–*C* behavior of EMC in this study. *P*–*V*–*T*–*C* model characterizes the relationship between specific volume and degree of cure of EMC under different pressure and temperature conditions. The *P*–*V*–*T*–*C* relationship is shown in Fig. 4.

18 $$\:\frac{\mathrm{1}}{\mathrm{V}}\mathrm{\:=\:}\frac{\mathrm{1}}{\mathrm{V}_\mathrm{uncured}}\cdot\left(\mathrm{1}-\mathrm{C}\right)\mathrm{+\:}\frac{\mathrm{1}}{\mathrm{V}_\mathrm{cured}}\cdot\mathrm{C}$$

19 $$\:\mathrm{V}_{\mathrm{uncured}\mathrm{/cured}}\mathrm{\:=\:}\mathrm{V}_\mathrm{0}\mathrm{[1}-\alpha \cdot \mathrm{ln(1+}\frac{\mathrm{P}}{\mathrm{B}}\mathrm{)]}$$

20 $$\:{{\rm{V}}_{\rm{0}}} = \left\{ {\begin{array}{*{20}{c}}{{{\rm{b}}_{{\rm{1s}}}}{\rm{ + }}{{\rm{b}}_{{\rm{2s}}}}\left( {{\rm{T}} - {{\rm{b}}_{\rm{5}}}} \right),{\rm{if}}\,{\rm{T}} \le {{\rm{T}}_{{\rm{trans}}}}}&{}\\{\:{{\rm{b}}_{{\rm{1L}}}}{\rm{ + }}{{\rm{b}}_{{\rm{2L}}}}\left( {{\rm{T}} - {{\rm{b}}_{\rm{5}}}} \right),{\rm{if}}\,{\rm{T > }}{{\rm{T}}_{{\rm{trans}}}}}&{}\end{array}} \right.$$

$\:\mathrm{V}$: specific volume; *C*: degree of cure; $\:V_\mathrm{0}$: specific volume at zero gauge pressure; $\:\alpha$: coefficient of thermal expansion; * P*: pressure; *B*: accounts for pressure sensitivity of the material; *b*_1*s*_, *b*_2*s*_, *b*_1*L*_, *b*_2*L*_: material constants; $\:b_\mathrm{5}$: transition temperature at zero gauge pressure; $\:\mathrm{T}_\mathrm{trans}$: glass transition temperature.

## Warpage analysis of RDL-first FOPLP

This section investigates the stress behavior of the redistribution layer (RDL) structure during RDL forming process, using Moldex3D, version 2025R1PR (https://ch.moldex3d.com/), as the primary simulation platform. A panel-level geometric model corresponding to the RDL-first process was constructed, with appropriate mesh sizing and material parameters defined. The simulation focuses on the evolution of thermal stresses as the structure cools from process temperature to room temperature. Key aspects of the analysis include residual stress distribution, warpage trends, and final warpage magnitude. The simulation results were further compared with experimental results to validate the accuracy and feasibility of the proposed modeling approach.

### Geometry of RDL-first structure

**Fig. 5:**
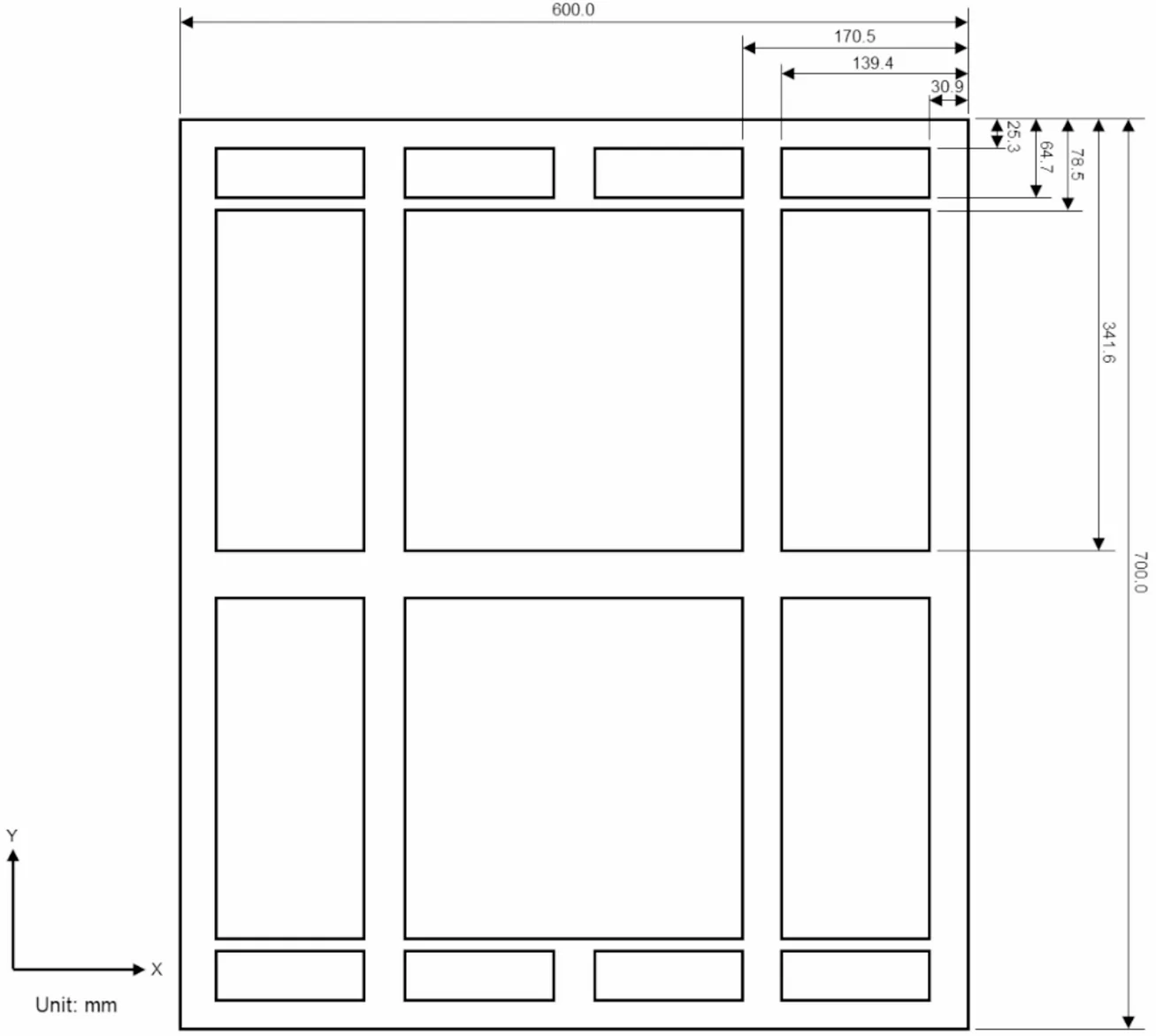
Top view of RDL-first panel geometry.

**Fig. 6:**
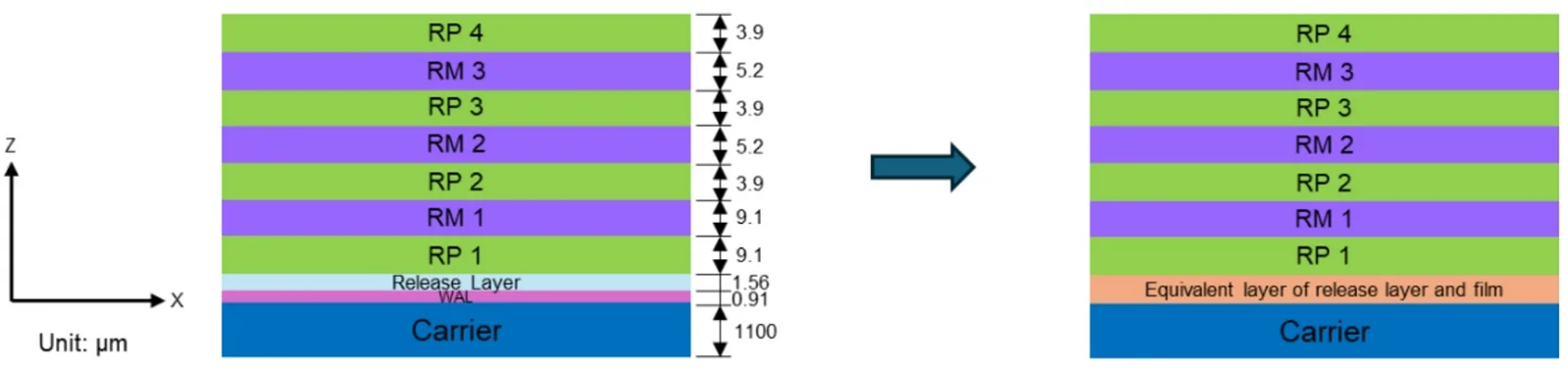
Cross-sectional view of the RDL-first panel geometry.

All geometric models used in this study are based on previous studies. Figure 5 presents the top view of the panel geometry. The regions labeled A, B, and C indicate areas with copper, while the remaining regions consist of dielectric material. The stacking sequence of the RDL and film structures is illustrated in Fig. 6, from bottom to top: carrier, silicon nitride (WAL), release layer, and RDL structures. The primary function of the WAL layer is to suppress warpage behavior. The RP and RM denote two types of RDL structures. The RP layer initially includes Cu vias and dielectric material; however, due to the negligible volume of the vias, they were omitted in the modeling process. The RM structure was modeled as an equivalent composite of copper and dielectric material. The copper contents of the RP 1 to RM 3 layers are listed in Table 1.

The release layer and WAL possess minimal thicknesses for the simulation analysis. Direct meshing of these layers would lead to significant mesh size ratio issues. To overcome this, equivalent modeling is applied to these two structures, improving mesh quality while maintaining computational accuracy, as shown in Fig. 6. The equivalent properties were calculated using a volume percentage method, which is widely adopted in RDL equivalent modeling to preserve global thermo-mechanical behavior. This treatment is intended for panel-level warpage prediction and maintains the dominant constraint effect of the thin layers while ensuring numerical stability.

**Table 1:** Cu area ratio for RDL.

Layer no.	Copper coverage (%)
RP 1	0
RM 1	26.6
RP 2	0
RM 2	10.43
RP 3	0
RM 3	5.39
RP 4	0

### Mesh model and boundary condition

In the RDL-first FOPLP simulation, geometric models with different X–Y plane mesh sizes were constructed, including mesh dimensions of 10 mm, 9 mm, and 8 mm. A subsequent mesh convergence study will be conducted to assess the influence of mesh refinement on simulation accuracy. The total number of elements in each mesh model is summarized in Table 2.

**Table 2:** Mesh size vs. number of elements.

Mesh size (mm)	Number of elements
10	251,580
9	315,117
8	568,748

**Fig. 7:**
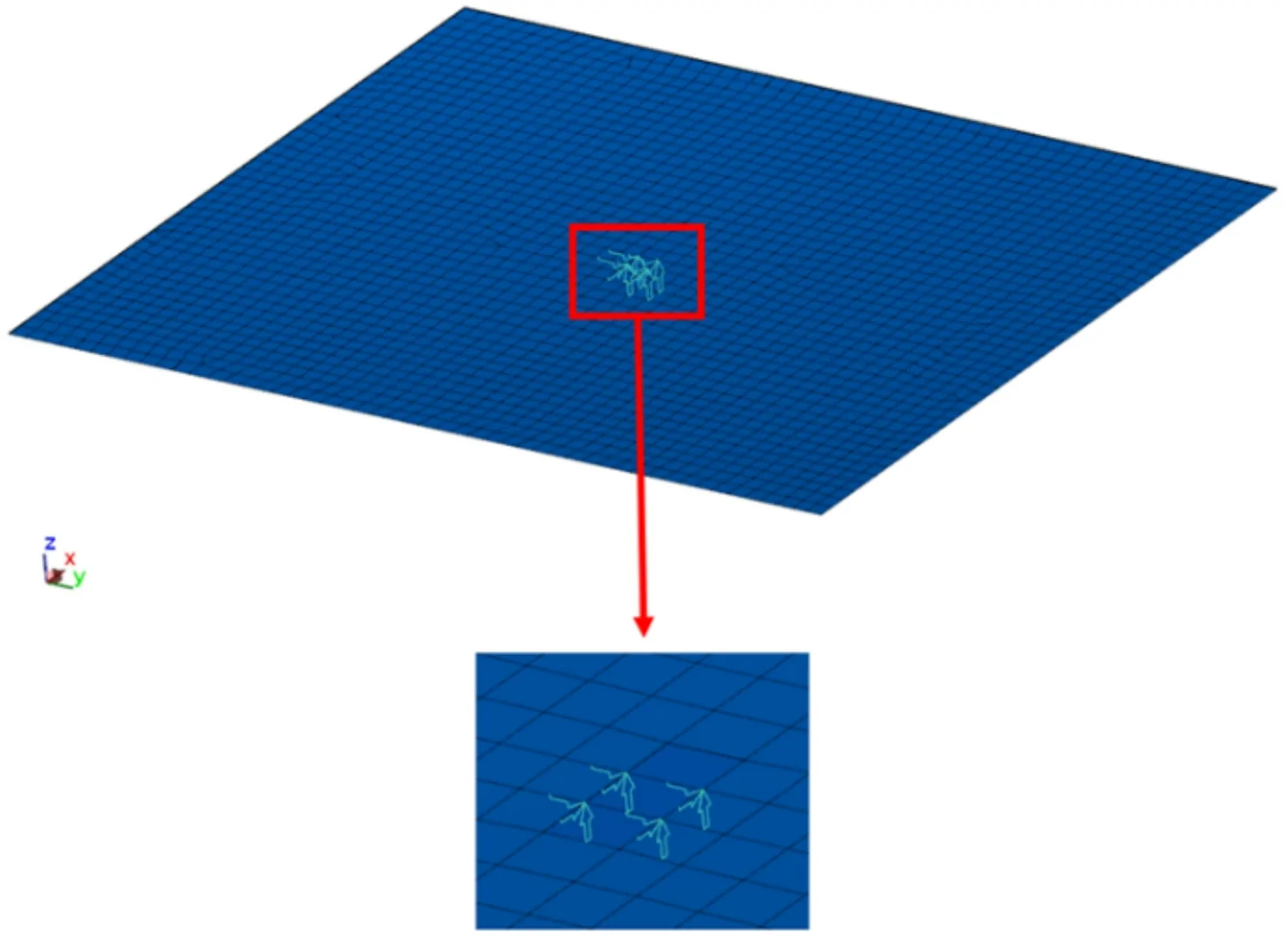
Boundary condition setting of RDL-first model.

To accomplish the RDL fabrication process on a perfectly flat platform, the boundary condition was defined by constraining the displacements in all three directions at the central bottom mesh node of the model, as illustrated in Fig. 7.

This constraint was applied to prevent rigid body rotation during the simulation without restricting the natural deformation of the structure.

### Material properties

The material properties, processing parameters, and packaging structures adopted in this study are derived from prior studies. For the thermal stress analysis of the redistribution layer (RDL), the main materials considered include copper, dielectric materials, release layer, silicon nitride (WAL), and various types of carriers.

The equivalent material modeling approach is adopted in this study to simplify the complex RDL structure. Modeling the actual copper routing patterns would require extremely fine meshes, leading to a significant increase in element count, computational cost, and potential numerical convergence issues. Therefore, the equivalent modeling approach provides a practical balance between computational efficiency and modeling feasibility.

To estimate the effective properties of these composite materials, many studies have developed different theoretical approaches based on mixture rules. In this work, a volume percentage method is adopted to compute the equivalent properties of multilayer composites. This approach, widely recognized in the literature, evaluates the overall behavior of a composite system by combining the intrinsic properties of each constituent material according to their respective volumetric ratios.

The equivalent Young’s modulus, coefficient of thermal expansion (CTE), and Poisson’s ratio are determined by the following formulations:

21 $$\:\mathrm{E}_\mathrm{eq}\text{}\mathrm{=\:}\mathrm{E}_\mathrm{A}\mathrm{V}_\mathrm{A}\text{}\mathrm{+\:}\mathrm{E}_\mathrm{B}\mathrm{V}_\mathrm{B}$$

22 $$\:\alpha_\mathrm{eq}\text{}\mathrm{=\:}\alpha_\mathrm{A}\mathrm{V}_\mathrm{A}\text{}\mathrm{+\:}\alpha_\mathrm{B}\mathrm{V}_\mathrm{B}$$

23 $$\nu_\mathrm{eq} = \nu_\mathrm{A}\mathrm{V}_\mathrm{A} + \nu_\mathrm{B}\mathrm{V}_\mathrm{B}$$

*E*_*eq*_, $\:\alpha\:$_*eq*_ and $\:\nu_\mathrm{eq}$: equivalences of Young’s modulus, CTE, and Poisson’s ratio for composite materials; *E*_*A*_, *E*_*B*_: Young’s modulus of materials A and B, respectively; $\:\alpha\:$_*A*_, $\:\alpha\:$_*B*_: CTE of materials A and B, respectively; $\:\nu\:$_*A*_, $\:\nu\:$_*B*_: Poisson’s ratio of materials A and B, respectively; *V*_*A*_, *V*_*B*_: volume percentages of materials A and B, respectively. The calculated equivalent material properties used in this study are summarized in Table 3.

**Table 3:** Material properties used in RDL-first warpage simulations.

Material	Young’s modulus (GPa)	Coefficient of thermal expansion (ppm/°C)	Poisson’s ratio	Density (g/cm^3^)
Copper	120	17	0.36	8.92
Dielectric	3.5	65	0.30	1.39
Release layer	3.54	3.78	0.23	0.9
WAL	112	2.9	0.24	3.2
RM 1 equivalent	34.23	33.88	0.316	3.694
RM 2 equivalent	15.30	37.60	0.306	2.543
RM 3 equivalent	9.40	38.76	0.303	2.184
Carrier materials				
Steel_1	155	7.8	0.26	8.2
Steel_2	155	8.5	0.26	8.2
Steel_3	155	10	0.26	8.2
Glass_1	77	3.8	0.22	2.4
Glass_2	70	7	0.22	2.4
Glass_3	70	8	0.22	2.4
Glass_4	70	9.1	0.22	2.4
Ceramic	370	7.8	0.30	2.4

### Process temperature settings

**Fig. 8:**
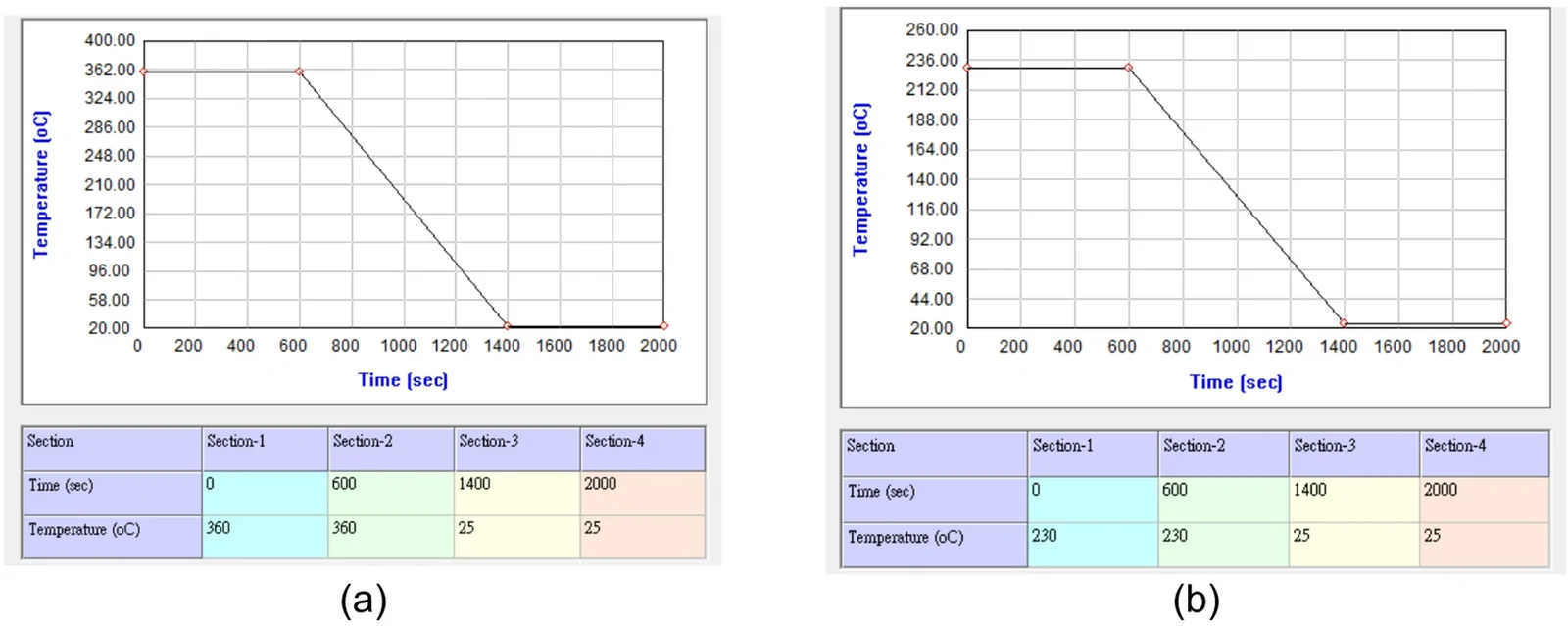
Temperature profile of simulations. (**a**) Equivalent layer. (**b**) RDL layers.

During the deposition of the silicon nitride layer (WAL), the process temperature reached 360 °C, while the release layer was applied at a lower process temperature of 230 °C. However, due to the extremely thin thickness of both layers, direct meshing without simplification would lead to poor mesh quality and convergence issues in the simulation. To address this, both structures were treated using an equivalent material modeling approach. For consistency and simplification, the process temperature of the equivalent structure was defined as 360 °C. The corresponding temperature profile for the WAL and release layer equivalent structure is illustrated in Fig. 8a. As for the RDL equivalent structure, the process temperature was set at 230 °C, and its temperature profile is shown in Fig. 8b.

In the equivalent structure of the RDL layer, the primary constituents are copper and dielectric materials. The process temperature of 230 °C corresponds to the stacking of dielectric layers. In contrast, the copper layers are electroplated at room temperature during fabrication. Therefore, applying a uniform process temperature of 230 °C to both materials in the equivalent model may introduce discrepancies between simulation results and actual behavior. To mitigate this, the equivalent CTE was calculated using Schapery’s energy-based method^5^, which minimizes the system’s strain energy while accounting for material heterogeneity. Subsequently, Lee^9^ extended this concept by employing a force equilibrium approach to derive an equivalent reference temperature that more accurately represents the thermal history of the RDL structure.

24 $$T_{\mathrm{eq,ref}}=\frac{V_A E_A \alpha_A T_{\mathrm{ref,A}}+V_B E_B \alpha_B T_{\mathrm{ref,B}}}{V_A E_A \alpha_A+V_B E_B \alpha_B}$$

*σ*_*A*_, *σ*_*B*_: normal stress components in materials A and B, respectively; *V*_*A*_, *V*_*B*_: volume percentages of materials A and B, respectively; *E*_*A*_, *E*_*B*_: Young’s modulus of materials A and B, respectively; $\:\alpha\:$_*A*_, $\:\alpha\:$_*B*_: CTE of materials A and B, respectively; *T*_*ref_A*_, *T*_*ref_B*_: reference temperature of materials A and B, respectively.

In previous modeling approaches, the equivalent reference temperature for thermal stress analysis was typically derived from a simplified single-layer RDL composite. However, directly applying this temperature to a multi-layer structure fails to capture the distinct thermal histories and mechanical responses of individual layers formed through sequential processing. This simplification often results in a systematic underestimation of warpage and introduces noticeable discrepancies compared to experimental measurements.

Moreover, although using the actual process temperature for initialization is physically justifiable, it frequently leads to an overprediction of deformation caused by thermal expansion. The present study proposes an average reference temperature to resolve the imbalance between underestimation and overestimation. This value is calculated as a weighted combination of the equivalent reference temperature from single-layer analysis and the real processing temperature. This refined initialization strategy mitigates previous modeling errors by accounting for differences in material properties and the continuity of thermal loading across layers. It enhances warpage simulations’ physical relevance and predictive accuracy for multilayer packaging structures.

25 $$T_{\mathrm{avg,ref}}=\frac{\left(\frac{V_A E_A \alpha_A T_{\mathrm{ref,A}}+V_B E_B \alpha_B T_{\mathrm{ref,B}}}{V_A E_A \alpha_A+V_B E_B \alpha_B}+T_{\mathrm{ref,A}}\right)}{2}$$

where material A is PI material and material B is copper material in this study.

The equivalent reference temperatures and average reference temperatures calculated from the six simulation analyses in the analysis process are shown in Table 4.

**Table 4:** Equivalent reference temperature settings used in simulation.

Simulation	Structures	Actual process temperature	Reference temperature	Average reference temperature
1.	Glass and equivalent layer	360 °C	360 °C	360 °C
2.	Glass, equivalent layer, and RP1	230 °C	230 °C	230 °C
3.	Glass, equivalent layer, RP1-2, and RM1	230 °C	68.03 °C	149.02 °C
4.	Glass, equivalent layer, RP1-3, and RM1-2	230 °C	125.36 °C	177.68 °C
5.	Glass, equivalent layer, RP1-4, and RM1-3	230 °C	159.62 °C	194.81 °C

### Analysis settings

This section first introduces the simulation tools and the settings for process temperature used in the thermal stress analysis. Subsequently, the results of the RDL process simulation are interpreted and validated.

To accurately simulate the temperature history and structural buildup during the RDL-first process of FOPLP, this study adopts the Element Birth and Death technique available in Moldex3D. This approach enhances the realism and accuracy of simulating multi-step processes.

Element Birth and Death is a widely used technique for modeling sequential material deposition in multi-stage processes. It enables the activation of structural elements layer by layer through actual manufacturing steps. When an element is “killed,” its stiffness matrix is reduced significantly (e.g., to 10^−6^ N/m), rendering it inactive in load and stress calculations. Conversely, when an element is “born,” it regains its original material properties and participates in the overall finite element computation.

**Fig. 9:**
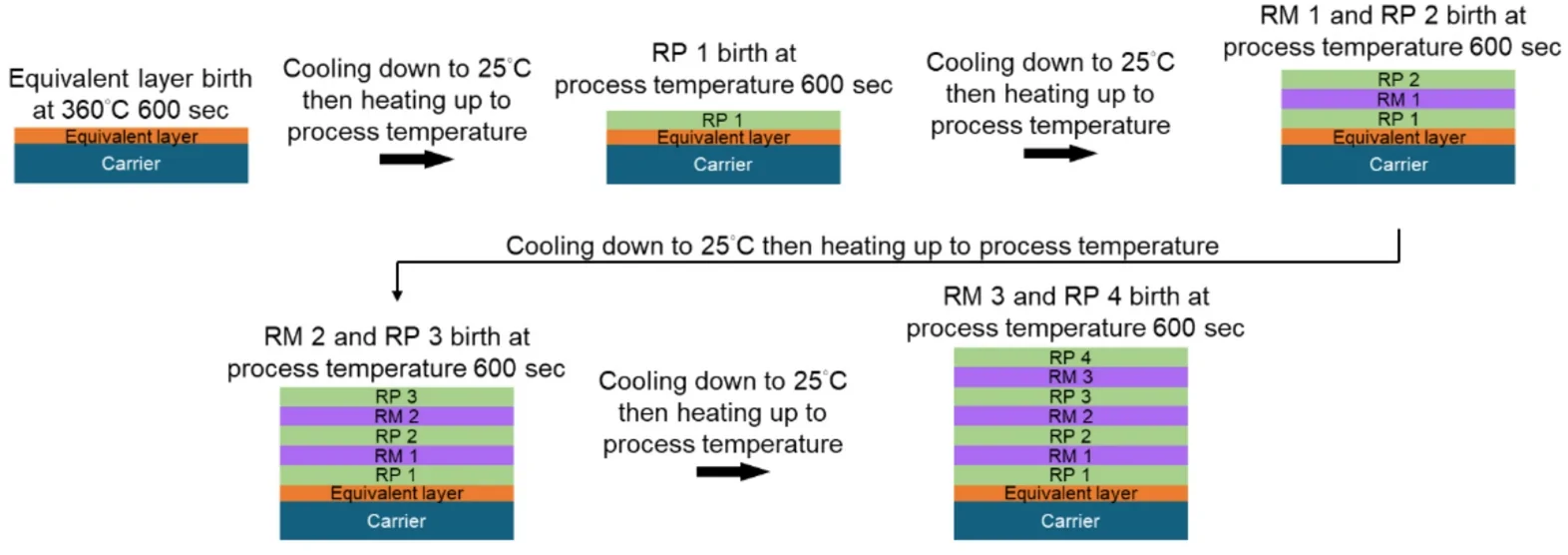
Simulation flow of RDL-first model.

This study simulates multiple stacking stages based on different geometric configurations to replicate real-world process conditions. As illustrated in Fig. 9, the simulation begins by analyzing thermal stress in RP 1, while the remaining layers are deactivated. Then, RM 1 and RP 2 elements are activated and analyzed. This process continues sequentially until the final RM 3 and RP 4 structure is fully simulated.

This simulation strategy effectively captures the layer-by-layer buildup under varying thermal and temporal conditions, yielding results that closely match actual processing behavior. As a result, the prediction of warpage and residual stress is significantly improved.

### Mesh convergence analysis

To ensure the stability and reliability of the simulation results, a mesh convergence study was conducted using three different mesh sizes (10 mm, 9 mm, and 8 mm). The study evaluated the impact of mesh resolution on diagonal warpage and maximum von Mises stress, along with the corresponding CPU time required for each case.

**Fig. 10:**
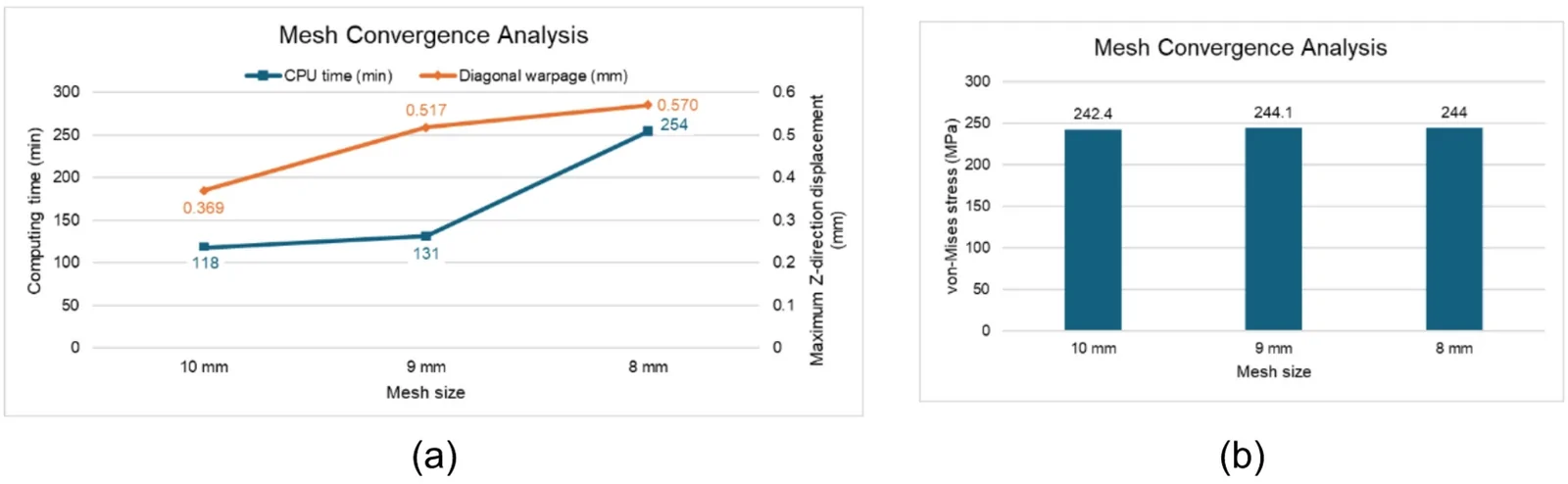
Results of mesh convergence analysis (**a**) warpage, (**b**) von-Mises stress.

As illustrated in Fig. 10, reducing the mesh size from 10 to 8 mm resulted in an increase in diagonal warpage from 0.369 to 0.57 mm, while the maximum von Mises stress showed signs of convergence, increasing only slightly from 242.4 to 244 MPa. With a 9 mm mesh size, the simulated warpage was approximately 0.517 mm, and the maximum von Mises stress reached 244.1 MPa, indicating that the results had approached convergence.

However, finer mesh sizes significantly increased the computational cost. CPU time rose from 118 min for the 10 mm mesh to 254 min for the 8 mm mesh. Taking both accuracy and computational efficiency into account, the 9 mm mesh size was adopted as the optimal setting for subsequent simulations.

It is worth noting that the maximum warpage predicted after the completion of the RP4 stacking stage was 0.517 mm, which closely aligns with the measured warpage of 0.460 mm, further validating the accuracy of the simulation model and confirming the appropriateness of the selected mesh resolution.

## Results and discussion of RDL-first FOPLP

### Results of RDL-first thermal stress analysis

**Fig. 11:**
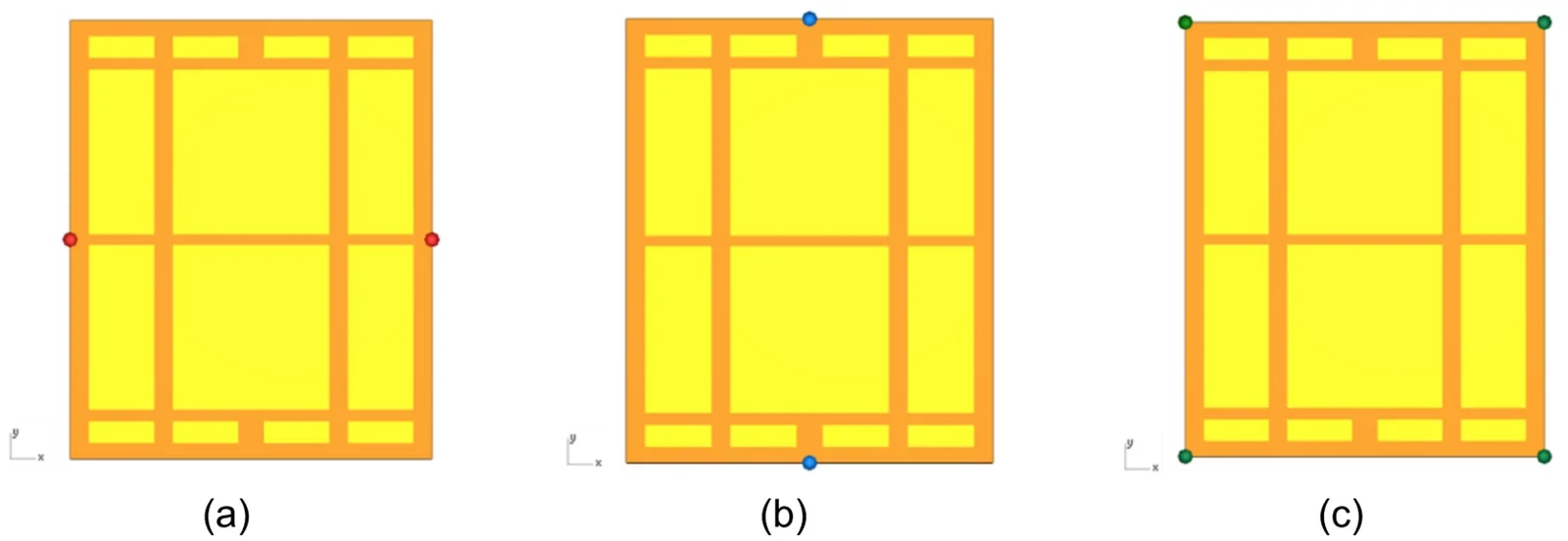
Schematic diagram of warpage definitions: (**a**) long-direction, (**b**) short-direction, (**c**) diagonal-direction.

Based on experiment measurements, panel warpage is classified into three directions: long-direction, short-direction, and diagonal, as illustrated in Fig. 11.

In the simulation, long-direction warpage is evaluated by observing the displacement variation along the centerline of the package structure in the long-side direction. Short-direction warpage is assessed by analyzing the deformation trend along the short-side direction. Meanwhile, diagonal warpage focuses on the vertical (Z-direction) deformation at the four corners of the panel.

This three-directional classification provides a comprehensive depiction of the package’s deformation behavior under multi-directional thermal and process-induced effects. It also serves as a critical reference for simulation validation and material selection. By categorizing and analyzing warpage in this manner, the study offers a more concrete understanding of potential warpage risks during actual manufacturing processes.

To begin the simulation, a glass carrier with a verified coefficient of thermal expansion of 3.8 ppm/°C was selected based on experimental validation. Warpage behavior was analyzed along the long-direction, short-direction, and diagonal directions. These simulations aimed to investigate the influence of different temperature initialization strategies on warpage prediction. All simulations utilized a mesh size of 9 mm and incorporated a sequential element activation approach to replicate the real stacking process.

**Fig. 12:**
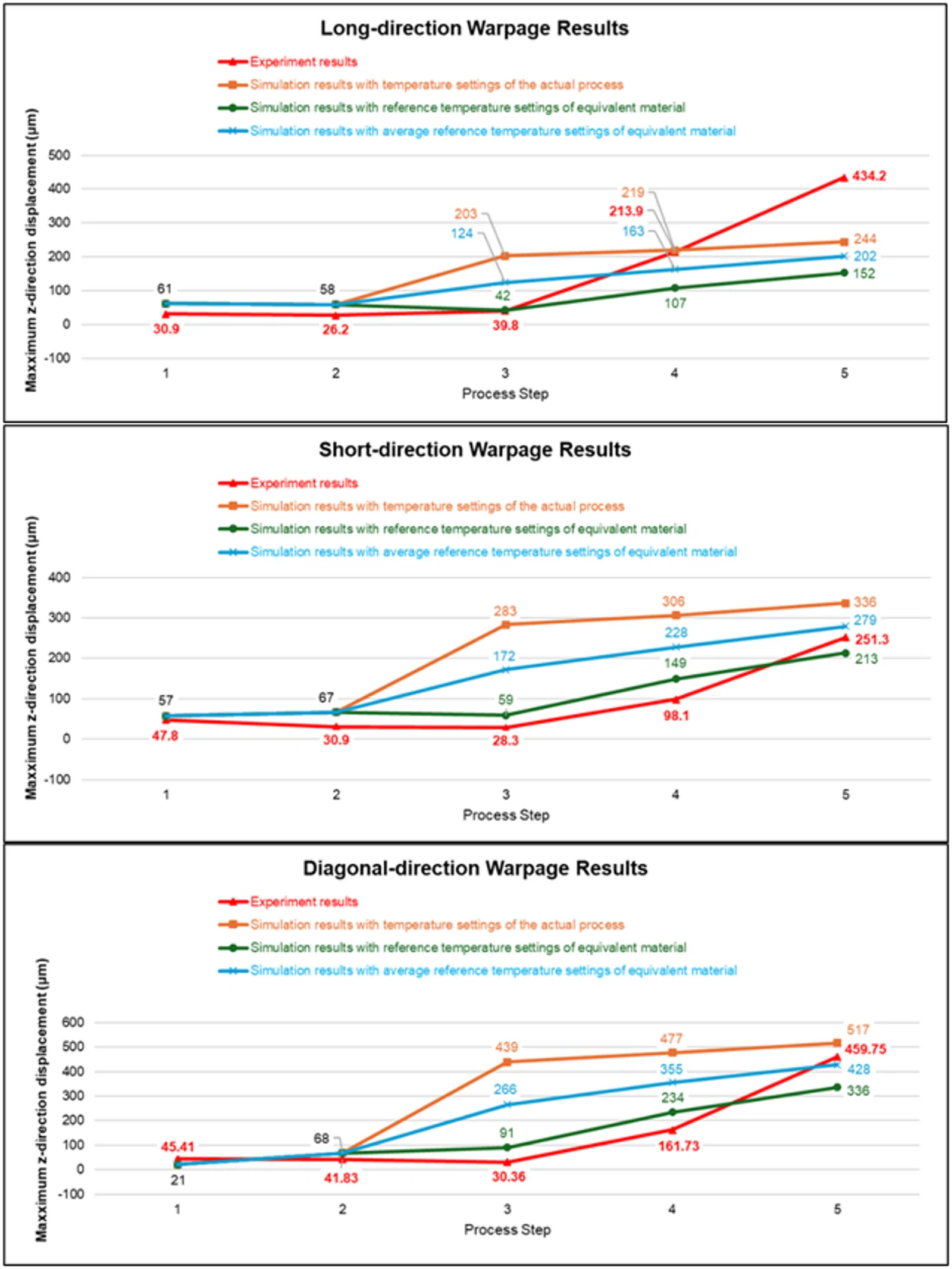
Results of warpage (**a**) long-direction, (**b**) short-direction, (**c**) diagonal-direction.

As shown in Fig. 12a, the simulation results for long-direction warpage revealed considerable deviations from experimental measurements across all temperature setting approaches. In contrast, the short-direction warpage results, illustrated in Fig. 12b, indicated that simulations initialized with the actual process temperature significantly overpredicted deformation, while the equivalent reference temperature underestimated it.

The diagonal warpage trends, presented in Fig. 12c, showed that using the actual process temperature and average reference temperature led to a progressive increase in simulated warpage from Step 1 through Step 5, closely following the experimental trend. At Step 5, the predicted warpage for actual process temperature and average reference temperature was 0.517 mm and 0.428 mm, respectively, which aligned well with the measured value of 0.459 mm. In comparison, simulations using the equivalent reference temperatures resulted in a lower predicted warpage of 0.336 mm, which significantly underestimated the actual deformation. Overall, the average reference temperature showed the most accurate prediction among the three methods (Table 5).

**Table 5:** Comparison of experimental and simulation warpage for different direction.

Maximum warpage (µm)							
Direction	Experiment	Reference temperature	Error	Average reference temperature	Error	Actual process temperature	Error
Long-direction	434.2	152	64.99%	202	53.47%	244	43.80%
Short-direction	251.3	213	15.24%	279	11.02%	336	33.70%
Diagonal-direction	459.75	336	26.91%	428	6.90%	517	12.45%
		Average	35.71%	Average	23.80%	Average	29.98%

This study further investigates the von Mises stress distribution in a fan-out panel-level package (FOPLP) with an RDL-first process utilizing a glass carrier with a CTE of 3.8 ppm/°C. The aim is to evaluate how different temperature initialization strategies affect the accuracy of stress predictions. Simulations were conducted using a multilayer structural model with a mesh size of 9 mm, focusing on both the stress distribution and the location of peak stress.

**Fig. 13:**
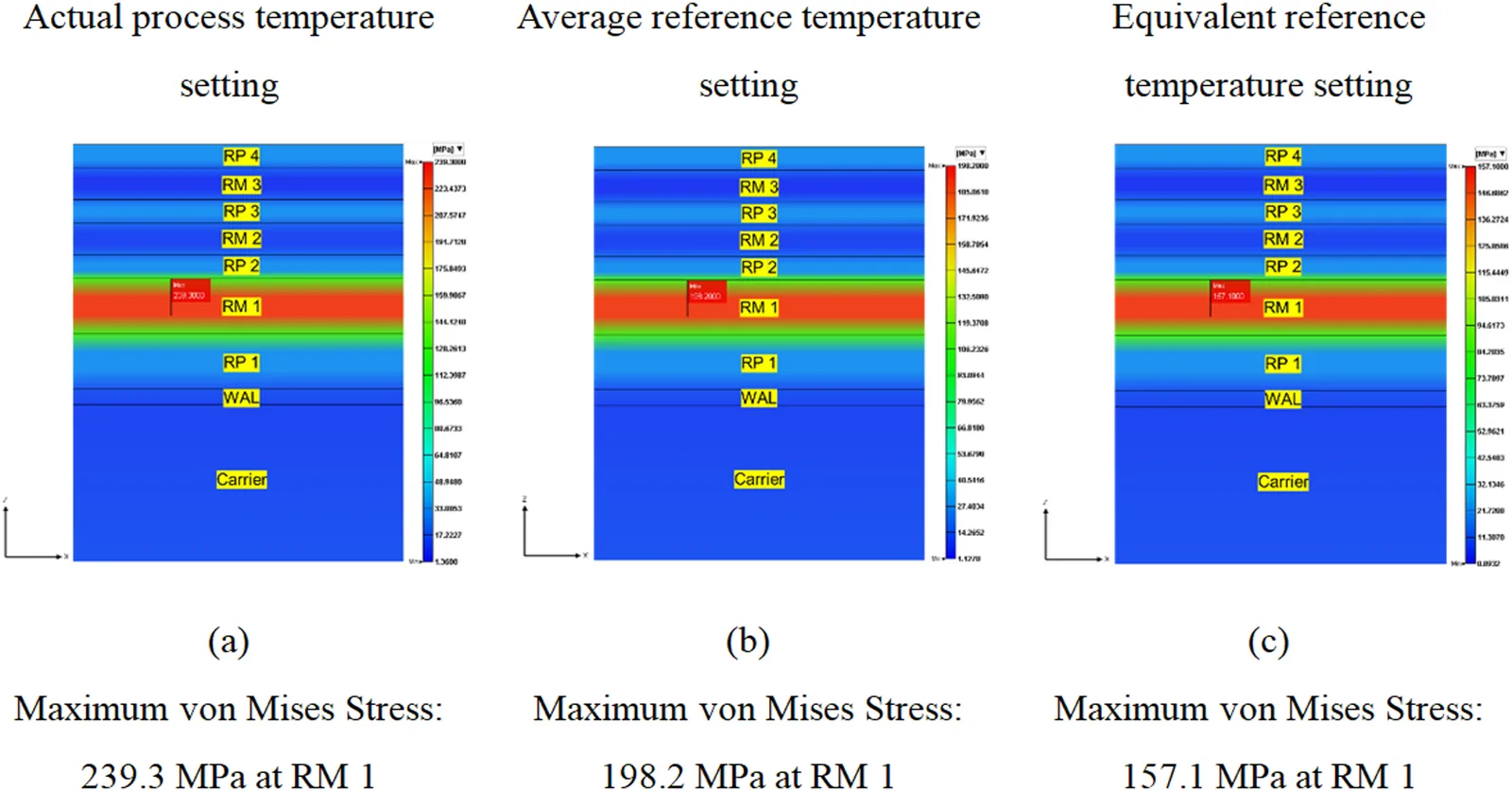
Results of maximum von Mises stress. (**a**) Simulation with actual process temperature setting. (**b**) Simulation with average reference temperature setting. (**c**) Simulation with equivalent reference temperature setting.

As shown in Fig. 13, under the simulation condition where the actual process temperature was applied, the highest von Mises stress occurred in the RM 1 layer, reaching 239.3 MPa. This value was significantly higher than those obtained using the average reference temperature (198.2 MPa) and the equivalent reference temperature (157.1 MPa). Given the current lack of direct experimental methods for measuring internal residual stress in packaging structures, the predicted maximum stress is primarily used as a reference but remains valuable for identifying potential failure locations.

Notably, regardless of the temperature setting used, the maximum stress consistently appeared in the RM 1 layer. In simulation settings, RM layers are modeled as equivalent material of copper and dielectric. A higher copper coverage increases the layer’s effective Young’s modulus and shifts its effective CTE closer to that of copper. In structure, RM 1 exhibits the highest copper coverage among the RM layers. Consequently, the mismatch in the coefficient of thermal expansion (CTE) between the RM 1 layer and its adjacent dielectric layers becomes more pronounced during cooling from the process temperature, resulting in larger von Mises stress. These results align with practical observations where failures frequently occur in the RDL layers. This consistency indicates that the simulation model possesses good physical correspondence and further confirms RM 1 as a critical location under thermomechanical loading conditions.

In summary, if the objective is to replicate actual process behavior and perform reliability risk assessments, adopting the average reference temperature in simulations effectively prevents underestimation of both warpage and stress, thereby enhancing the accuracy and reliability of the simulation results.

### Simulation results between different carriers with average reference temperature settings

Following the establishment of a simulation workflow validated against experimental results, this study further investigates the influence of carrier materials with different CTE on the warpage behavior of FOPLP structures. Various commonly used carrier materials were considered, including glass carriers with varying CTE values (Glass_1 to Glass_4), steel carriers (Steel_1 to Steel_3), and a ceramic carrier. All simulations were conducted under consistent geometric configurations and identical process conditions to ensure fair and representative comparisons.

As summarized in Table 6, the simulation results reveal that the choice of carrier material has a pronounced effect on the maximum warpage. Carriers with higher CTE (e.g., Glass_2 to Glass_4 and Steel_3) exhibit larger warpage magnitudes than those with lower CTEs. For instance, within the glass group, increasing the CTE from 3.8 ppm/°C to 9.1 ppm/°C leads to a corresponding rise in maximum warpage from 0.4283 to 0.4714 mm, indicating a consistent correlation with thermal expansion behavior. Similarly, within the steel group, the carrier with a CTE of 7.8 ppm/°C (Steel_1) exhibited a warpage of 0.2665 mm, which is lower than the 0.2823 mm observed for the carrier with a CTE of 10 ppm/°C (Steel_3).

Of particular note, the ceramic carrier (CTE: 7.8 ppm/°C) exhibits the smallest warpage value of 0.1711 mm among all tested materials, despite having a CTE similar to Steel_1. This reduction in warpage may be attributed to the ceramic’s significantly higher Young’s modulus and superior thermal stability, which enhance its resistance to deformation under thermal-mechanical loading.

In summary, the combination of CTE and Young’s modulus is critical in determining the overall warpage behavior of fan-out packages. Selecting a carrier with optimal thermo-mechanical properties is essential for minimizing process-induced deformation and improving the overall reliability and yield of advanced packaging. The insights obtained from this study provide a physics-based reference for material selection and structural design in future FOPLP applications (Table 7).

**Table 6:**
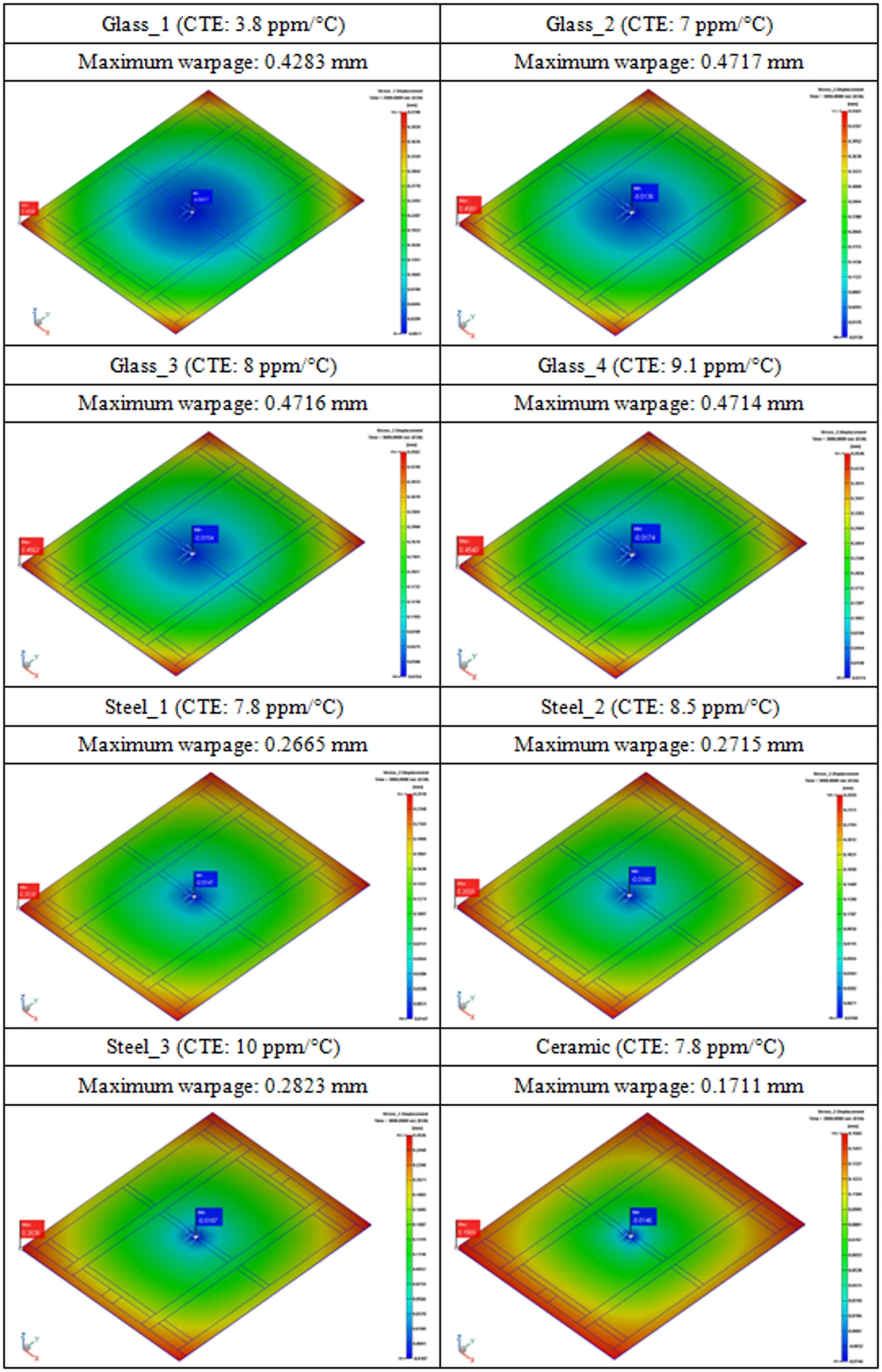
Comparison of RDL-first warpage simulation results with different carriers.

**Table 7:** Summary of RDL-first FOPLP warpage simulation results.

Carrier type	Young’s modulus (GPa)	Coefficient of thermal expansion (ppm/°C)	Maximum warpage (mm)			Maximum von Mises stress (MPa)
			Long direction	Short direction	Diagonal direction	
Glass_1	77	3.8	0.202	0.279	0.428	198.2 (RM 1)
Glass_2	70	7	0.252	0.332	0.454	192.8 (RM 1)
Glass_3	70	8	0.261	0.340	0.453	190.3 (RM 1)
Glass_4	70	9.1	0.272	0.349	0.450	187.6 (RM 1)
Steel_1	155	7.8	0.180	0.220	0.250	176.1 (RM 1)
Steel_2	155	8.5	0.189	0.228	0.270	174.4 (RM 1)
Steel_3	155	10	0.207	0.247	0.261	170.8 (WAL)
Ceramic	370	7.8	0.143	0.164	0.170	204.8 (WAL)

## Warpage analysis of molding-first FOPLP

This chapter presents the flow and structural warpage analysis of the molding-first process. A symmetric model was constructed using Moldex3D software to simulate the warpage behavior of fan-out panel-level packaging (FOPLP) after compression molding and subsequent carrier debonding. The simulation accounts for both the cure shrinkage induced by epoxy molding compound (EMC) during the compression process and the thermal contraction resulting from mismatches in material properties. The residual stress generated during molding was incorporated into the structural analysis, which employs the element birth and death technique to simulate the debonding sequence. For validation, the structure using a steel carrier was compared against experimental results. Based on the established methodology, this study further evaluates the warpage behavior of alternative carrier materials with varying properties.

### Geometry of molding-first FOPLP

**Fig. 14:**
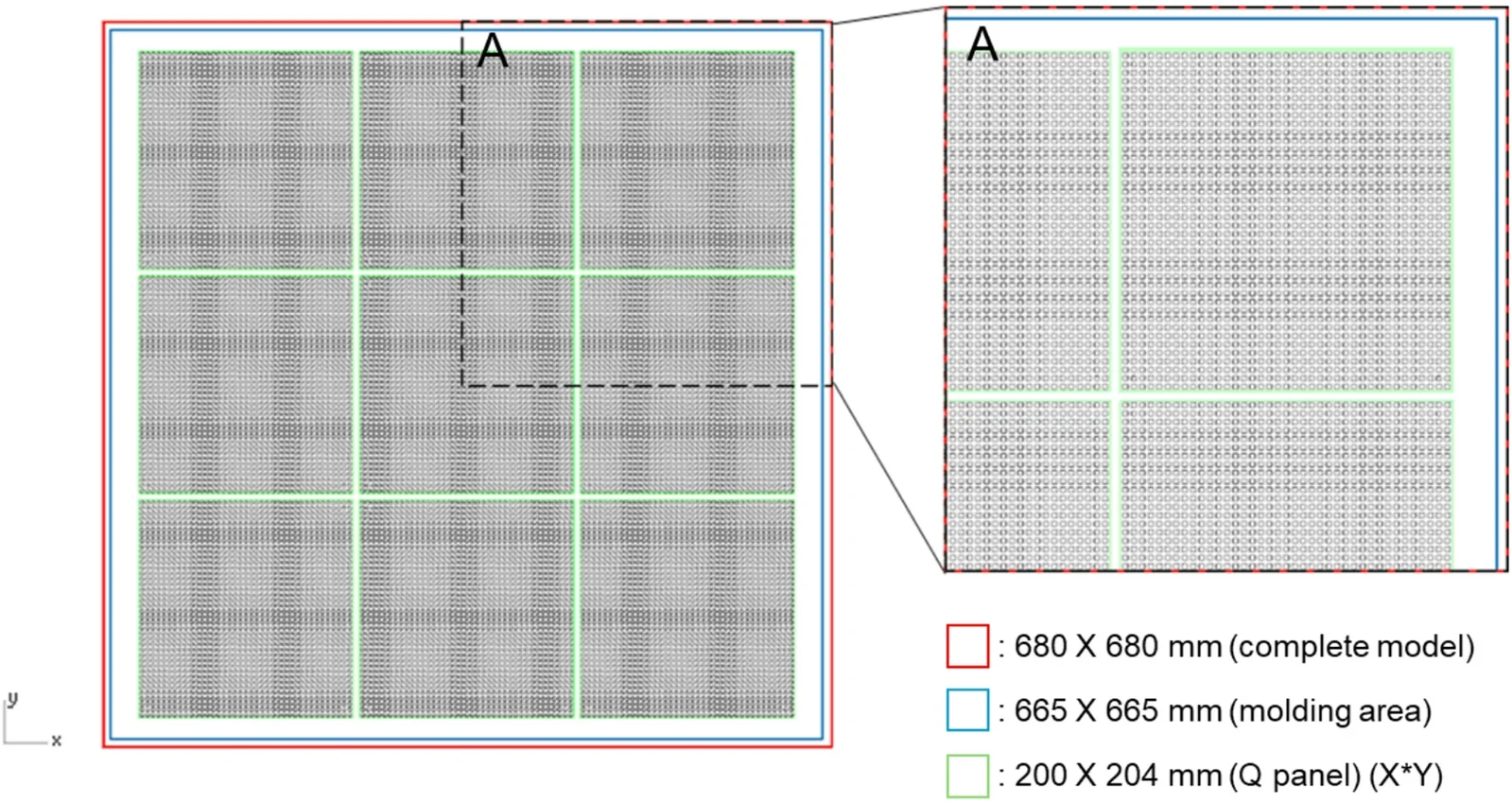
Top view of global molding-first model.

The geometrical models discussed in this chapter are derived from previous studies. A quarter-symmetry finite element model was constructed to simulate the warpage and stress behavior of the panel during the compression molding and carrier removal stages in the molding-first process, as shown in Fig. 14.

**Fig. 15:**
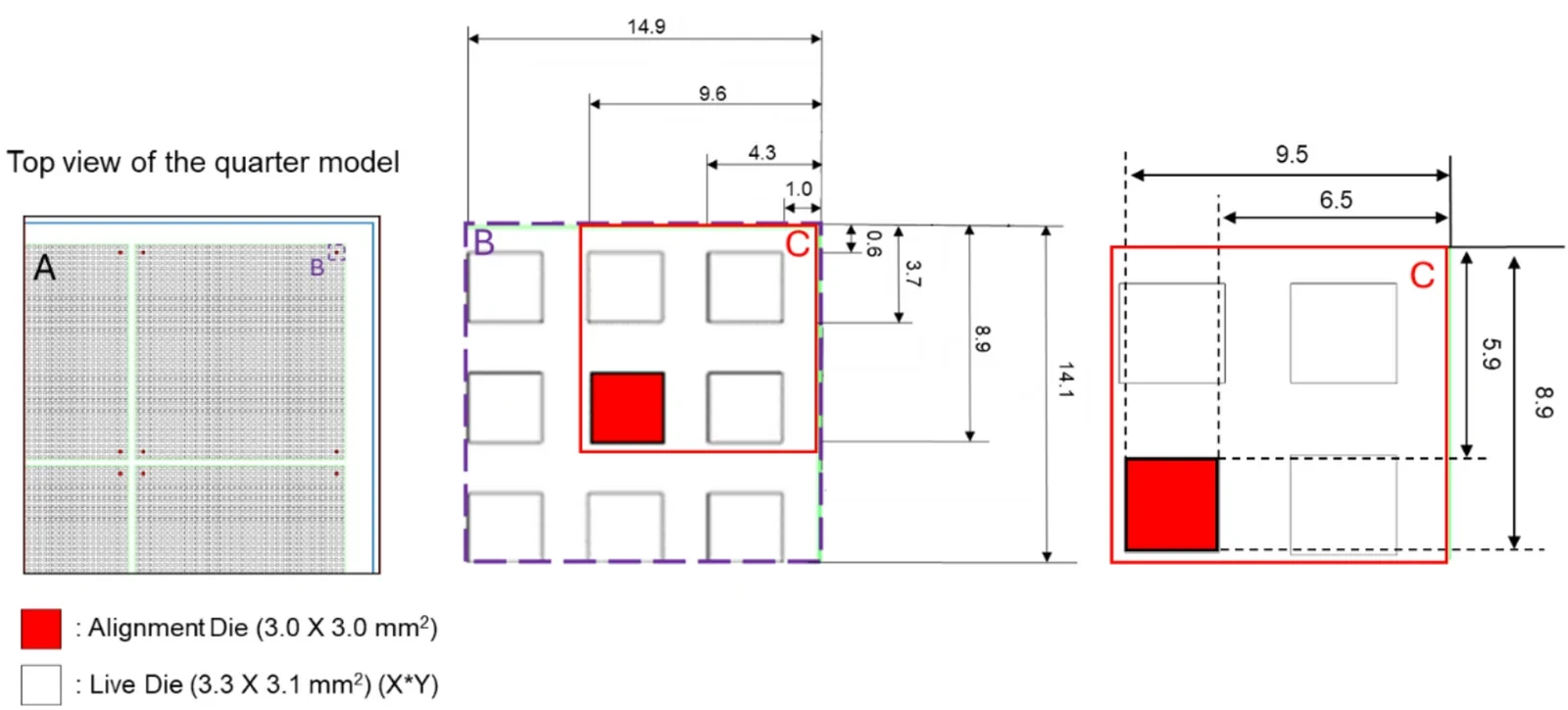
Top view of quarter-symmetry molding-first model.

The entire panel consists of nine Q-panels. Each Q-panel has 1556 standard dies and four alignment dies, as illustrated in Fig. 15. Using a quarter-symmetry model significantly reduces computational demands while preserving the geometric representativeness of the die arrangement. This setup enhances the model’s ability to accurately capture filling behavior and warpage during the simulation.

**Fig. 16:**
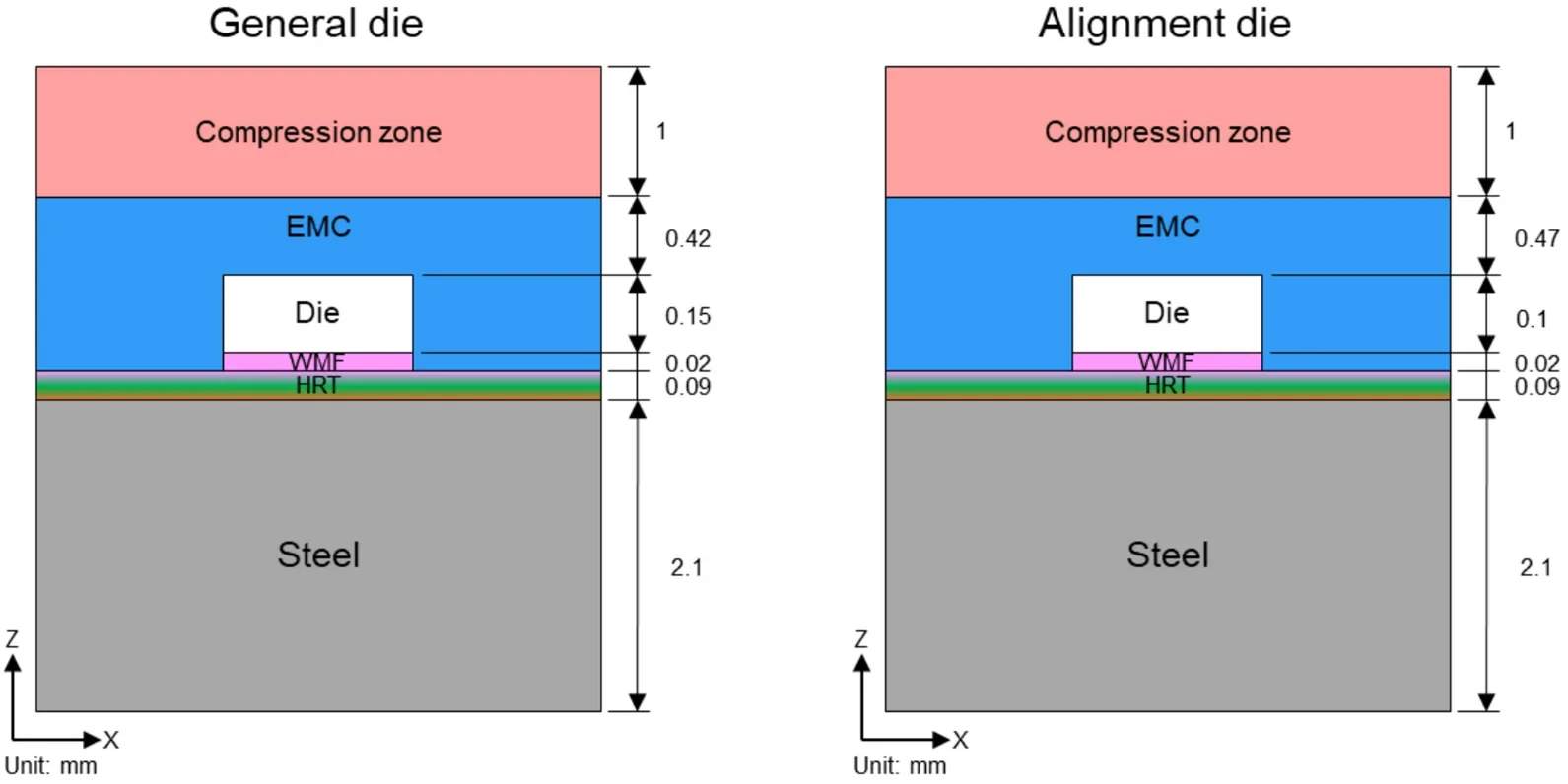
Cross-sectional view of molding-first model.

A schematic cross-sectional view of the structure is provided in Fig. 16. From bottom to top, the components include carrier, heat release tape (HRT), dielectric film (WMF), dies, and epoxy molding compound (EMC) serving as the encapsulant.

**Fig. 17:**
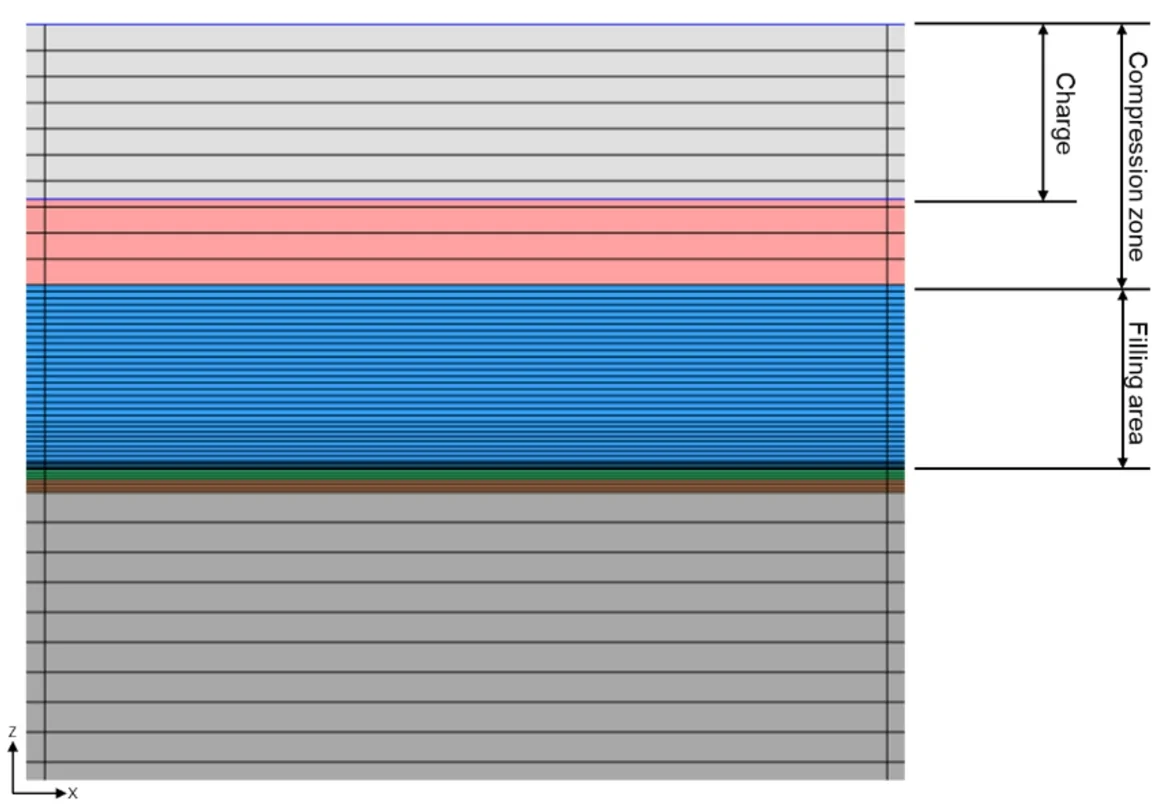
Schematic diagram of compression zone and charge.

The molding process applied in this simulation is compression molding. A compression zone and a pre-filling material charge are incorporated into the model to replicate the actual process where the structure is heated, moved, and compressed until complete curing is finished. These features, shown in Fig. 17, facilitate the realistic simulation of compression behavior under thermal and mechanical loading.

### Materials properties

The material properties adopted in this study are based on previous studies. All materials were characterized under room temperature conditions. In addition, the properties of the epoxy molding compound (EMC) were provided by CoreTech System Co., Ltd. and incorporated into the simulation using a combination of viscosity model, cure kinetics model, and *P–V–T–C* model to reflect its thermomechanical behavior. The mechanical material properties used in the simulation are summarized in Table 8.

**Table 8:** Material properties used in molding-first warpage simulations.

Material	Young’s modulus (GPa)	Coefficient of thermal expansion (ppm/°C)	Poisson’s ratio	Density (g/cm^3^)
Die (Si)	131	2.8	0.26	2.329
EMC	25	8.8	0.3	1.996
WMF	10	15	0.3	0.9
HRT	0.108	3	0.49	1.5
Steel_1	155	7.8	0.26	8.2
Steel_2	155	8.5	0.26	8.2
Steel_3	155	10	0.26	8.2
Steel_4	210	12	0.3	7.87
Glass_1	70	7	0.22	2.4
Glass_2	70	8	0.22	2.4
Glass_3	70	9.1	0.22	2.4
Glass_4	70	10	0.22	2.4
Ceramic	370	7.8	0.30	2.4

### Process parameter settings

The relevant processing parameters and their definitions are introduced in this section to simulate the compression molding process adopted in this study. All parameters are derived from previous studies.

1. Compression time: This refers to the total duration required for the resin to be fully compressed from the top of the compression zone into the mold cavity and to complete the subsequent curing stage. This duration excludes the pre-heating time and is set to 300 s.
2. Compression gap: This represents the thickness of the compression zone and is defined as 1 mm.
3. Compression speed: This indicates the rate at which the compression is carried out. It can be configured as a time-dependent variable; however, this study applies a constant speed of 0.15 mm/s millimeters per second.
4. Compression force: This denotes the maximum force exerted during the compression process, which is set to 300 tons-force.
5. Resin temperature: This is the initial temperature of the molding compound before the onset of the pre-heating stage, defined as 90 °C.
6. Mold temperature: This refers to the temperature of the mold cavity. It is typically selected based on the reaction kinetics model. In this study, the mold temperature is set to 175 °C.
7. Initial conversion: This parameter represents the initial degree of cure of the molding compound. Since the resin is generally stored in a freezer or low-temperature environment before processing, it is brought to room temperature before molding. Thermal exposure may induce premature curing. For this simulation, the initial conversion is assumed to be 0% after the resin is left at 25 °C.
8. Pre-heating time: Prior to compression molding, the epoxy molding compound must be pre-heated from room temperature to a lower-viscosity state to facilitate flow and cavity filling. This duration affects the resin’s initial degree of cure and viscosity before compression. The pre-heating time is set to 20 s.

### Boundary conditions settings

**Fig. 18:**
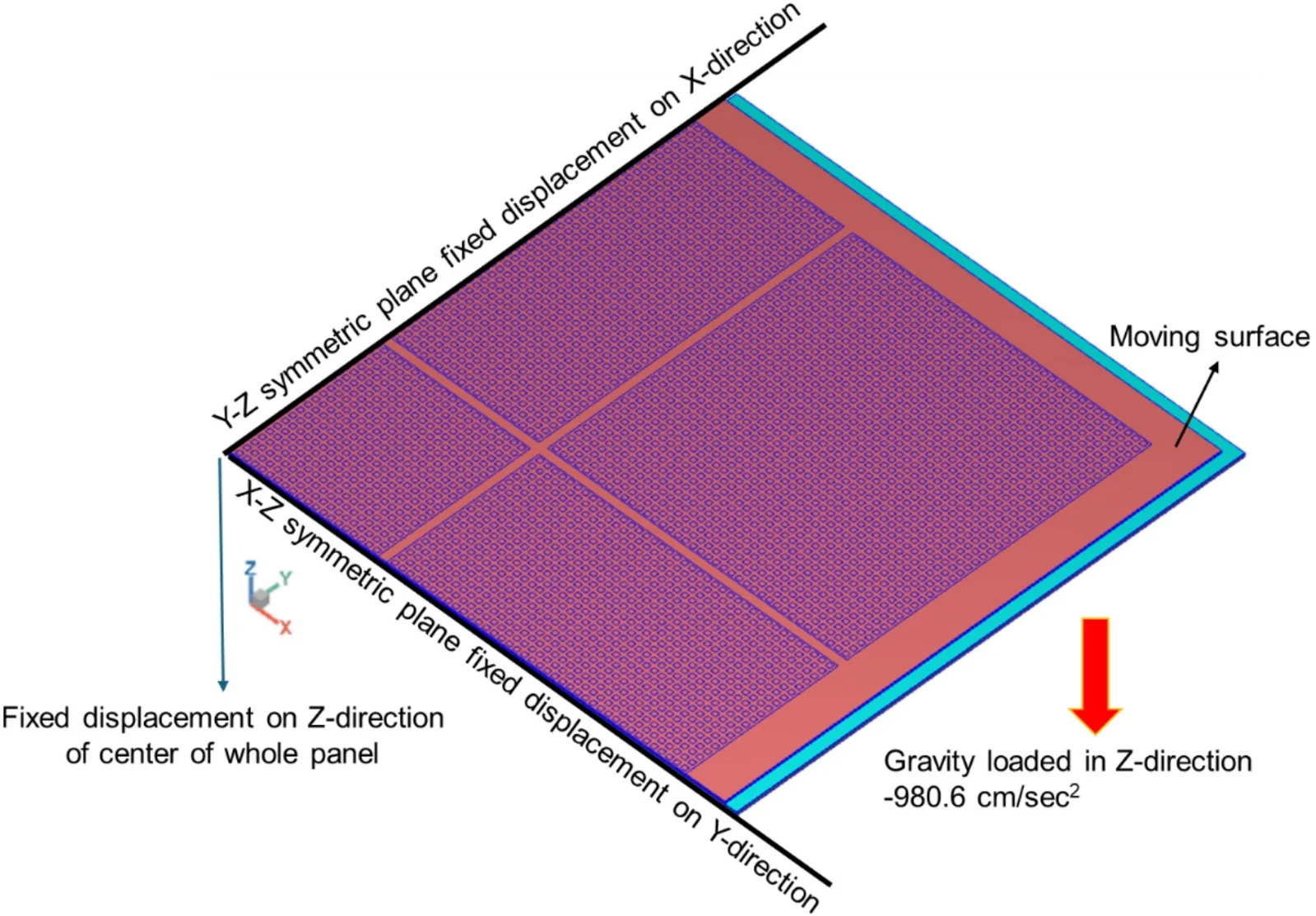
Boundary condition setting of molding-first model.

To accurately replicate the compression molding process of the molding-first packaging structure, this study defined the moving surface above the compression zone, as illustrated in Fig. 18. The moving surface represents the upper boundary that drives the compression of the EMC during the filling stage. This setup allows the moving surface to move across the entire height of the compression zone, pushing the EMC downward and thereby emulating the material flow seen in actual compression molding conditions.

In order to accurately capture the filling and warpage behavior of the package structure during the compression molding and carrier removal stages in the molding-first process, appropriate boundary and symmetry constraints were applied to a quarter-symmetry model. Symmetry conditions were imposed along the x- and y-axis planes to preserve the geometric consistency of the structure.

In addition to basic displacement constraints, gravitational effects were also incorporated to enhance the accuracy of warpage prediction. The gravitational acceleration was set to − 980.6 cm/s^2^, corresponding to Earth’s gravity, to simulate the additional stresses and deformations induced by self-weight in the vertical direction. This consideration is particularly critical for large-area and multi-layer package structures, where the gravity effect can significantly affect warpage behavior.

### Analysis settings

In this study, Moldex3D was employed to perform a two-stage simulation corresponding to the compression molding and carrier removal phases, focusing on the thermo-mechanical behavior of the package. To accurately simulate material behavior transitions, the element birth and death technique was applied to model the addition and removal of critical material layers, while simultaneously capturing structural responses during temperature variations.

**Fig. 19:**
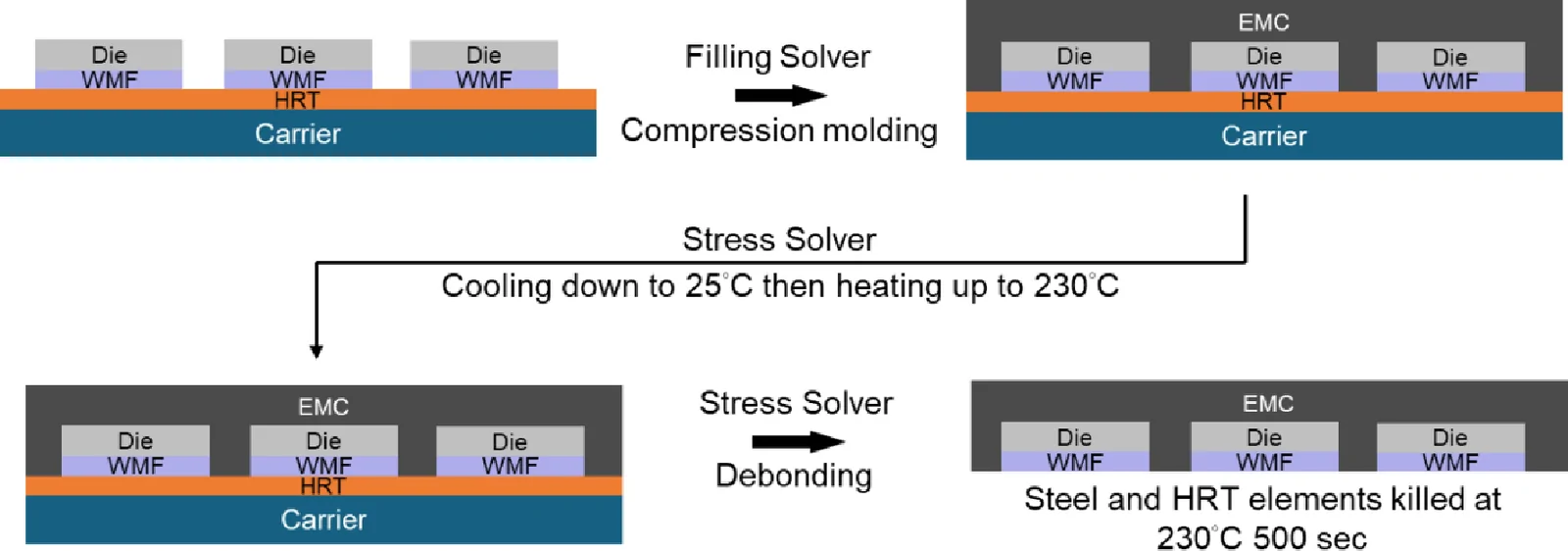
Simulation flow of molding-first FOPLP warpage analysis withelement birth and death technique.

As shown in Fig. 19, the model included components such as the carrier, heat release tape (HRT), dielectric film (WMF), and die. All elements were activated at the start of the simulation. The initial temperature was set to 175 °C, corresponding to the mold heating condition, followed by a cooling process to room temperature to analyze warpage and stress evolution induced by thermoset materials during curing and cooling.

In the carrier removal stage, the model accounted for the process condition in which the carrier and HRT were detached at 230 °C. In the simulation, elements representing steel and HRT were deactivated by assigning near-zero stiffness, thereby mimicking the structural release. After debonding, the model was cooled to room temperature to assess the final warpage condition.

Two separate thermal processes were defined to examine the effects of compression molding and carrier removal on structural deformation and stress distribution in the molding-first process. Warpage and residual stress variations under different thermal environments were analyzed.

**Fig. 20:**
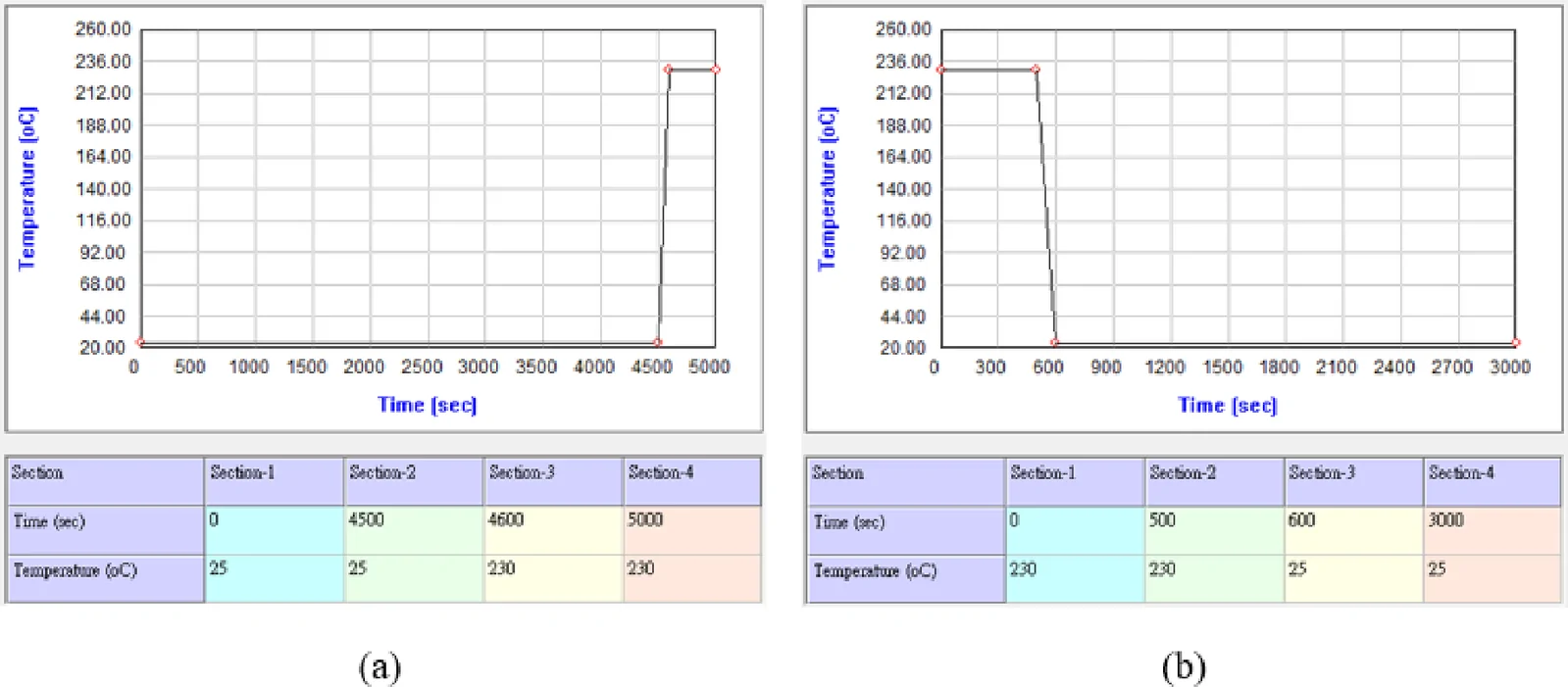
Temperature profile of simulations. (**a**) After molding (**b**) After debonding.

As illustrated in Fig. 20, the initial temperature in the compression molding phase was set to 175 °C, reflecting actual mold conditions. The panel was held at room temperature for 4500 s to replicate post-molding cooling in ambient conditions. This stage evaluated warpage and internal stress accumulation caused by thermal and curing shrinkage, serving as the baseline before debonding.

In the debonding phase, the initial temperature was set to 230 °C and maintained for 500 s before cooling to 25 °C. This stage simulated EMC shrinkage and stress release upon carrier removal. Since significant warpage changes occur during this phase, the simulation aimed to capture the final deformation state after processing and to evaluate the warpage control effectiveness of various carrier materials.

## Results and discussion of molding-first FOPLP

This study further explores the effect of carrier material properties on the warpage behavior after molding and debonding. As summarized in Table 9, various carriers with different CTE and Young’s modulus were evaluated, including steel, glass, and ceramic materials. All models were simulated under identical geometric and processing conditions to ensure consistent comparison and physical representativeness.

The ceramic carrier (CTE: 7.8 ppm/°C) exhibited the smallest deformation among all materials, showing warpage values of 1.158 mm after molding and 9.339 mm after debonding. Although its CTE is comparable to that of Steel_2, its substantially higher Young’s modulus provides superior dimensional stability and strong resistance to thermomechanical deformation, making ceramic a highly reliable material for large-panel packaging applications.

For the steel carrier group, a distinct correlation between CTE and warpage evolution was observed. Steel_1 (CTE: 7.8 ppm/°C) exhibited a warpage of 2.787 mm after molding, which decreased slightly to 1.815 mm for Steel_2 (CTE: 8.5 ppm/°C). When the CTE increased to 10 ppm/°C (Steel_3), the warpage value turned slightly negative, about − 0.858 mm, suggesting a near-balanced thermal stress distribution during molding. However, after carrier debonding, the release of residual stresses significantly amplified the deformation, reaching up to 9.453 mm for Steel_3. Among all steel materials, Steel_4 (CTE: 12 ppm/°C) exhibited the highest stiffness but also the greatest warpage variation, changing from − 2.366 mm after molding to 15.761 mm after debonding. These results indicate that steel carriers with higher stiffness and CTE are prone to stress-release amplification during the debonding stage.

Within the glass carrier group, increasing the CTE from 7 ppm/°C (Glass 1) to 10 ppm/°C (Glass 4) reduced the warpage after molding from 27.727 mm to 18.695 mm, whereas the warpage after debonding remained around 30 mm for all glass types. This observation suggests that although a higher CTE increases thermal strain, the relatively low Young’s modulus of glass dominates the deformation behavior, resulting in consistently large overall warpage regardless of CTE variations.

In addition, the significant increase in warpage observed after debonding can be attributed to the removal of the carrier, which acts as a primary structural constraint during the molding stage. Due to its substantially greater thickness and higher Young’s modulus, the carrier provides strong mechanical support that suppresses deformation. Once the carrier is removed, the overall structural stiffness is significantly reduced, leading to the release of residual stress accumulated during processing. This stress redistribution results in a pronounced amplification of warpage.

Overall, these results reveal that both CTE and Young’s modulus critically influence warpage behavior in molding-first processes. A carrier with moderate CTE and high stiffness, such as ceramic, can effectively minimize process-induced deformation and enhance structural reliability during large-panel packaging manufacturing (Table 10).

**Table 9:**
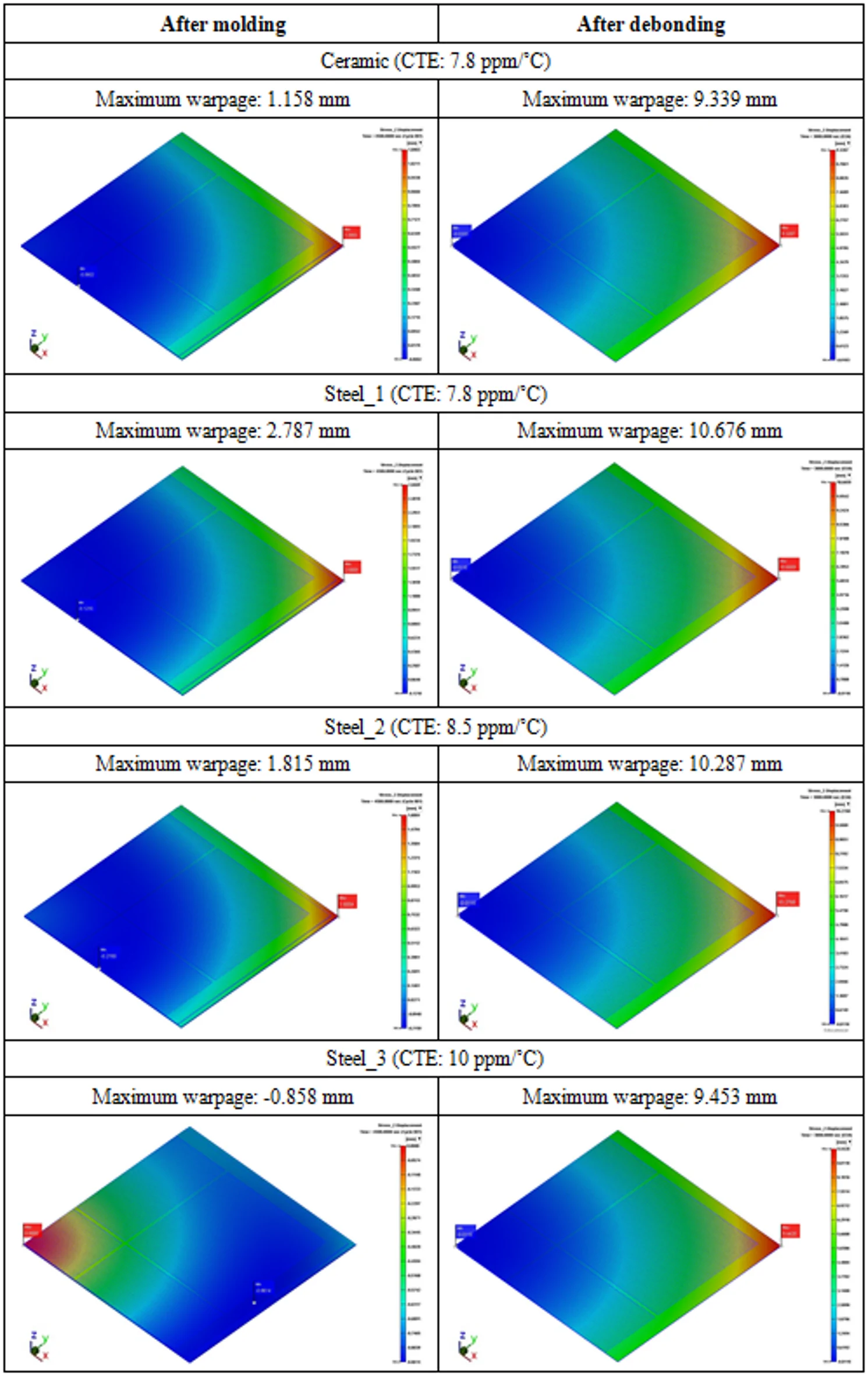

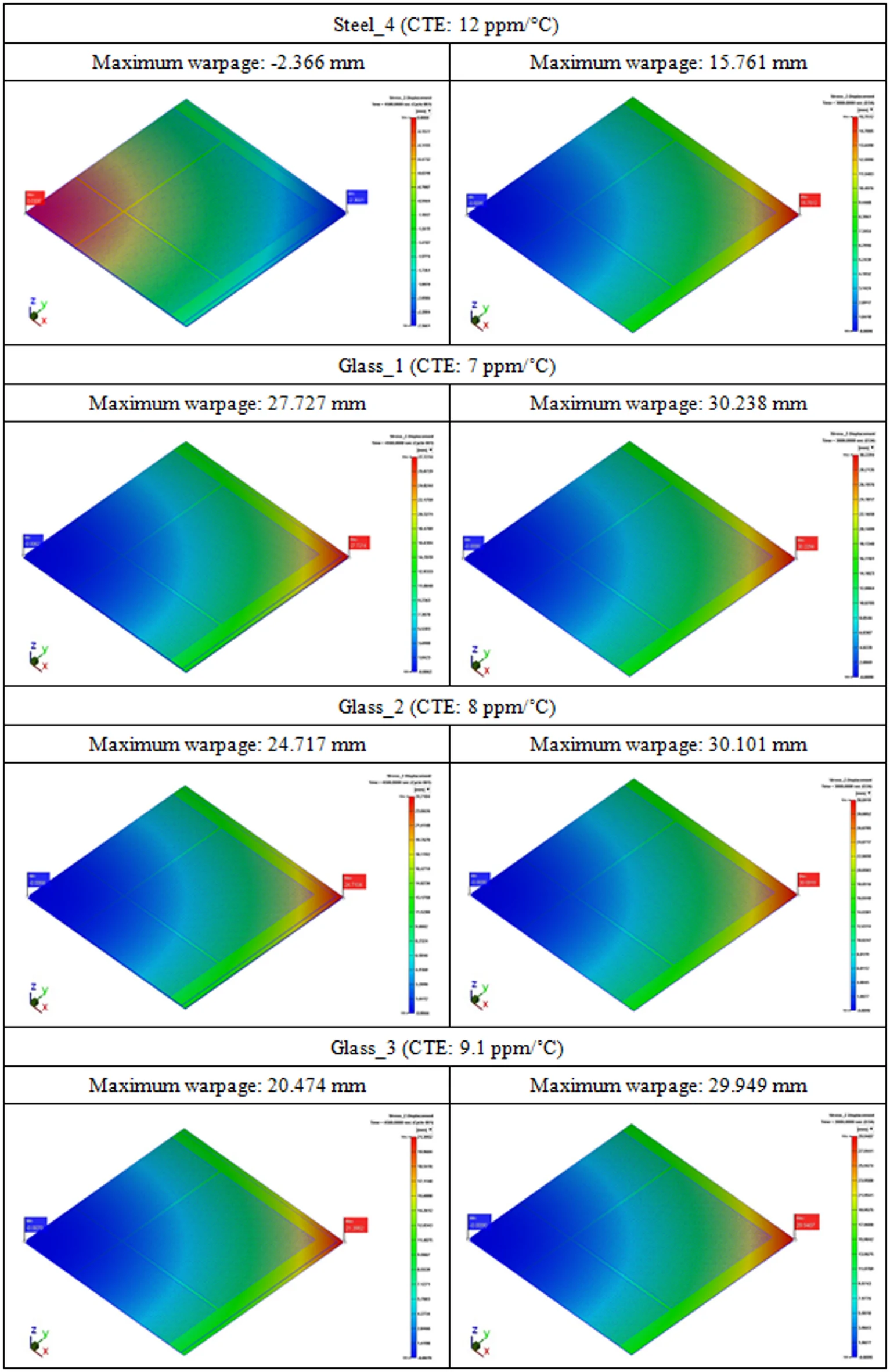

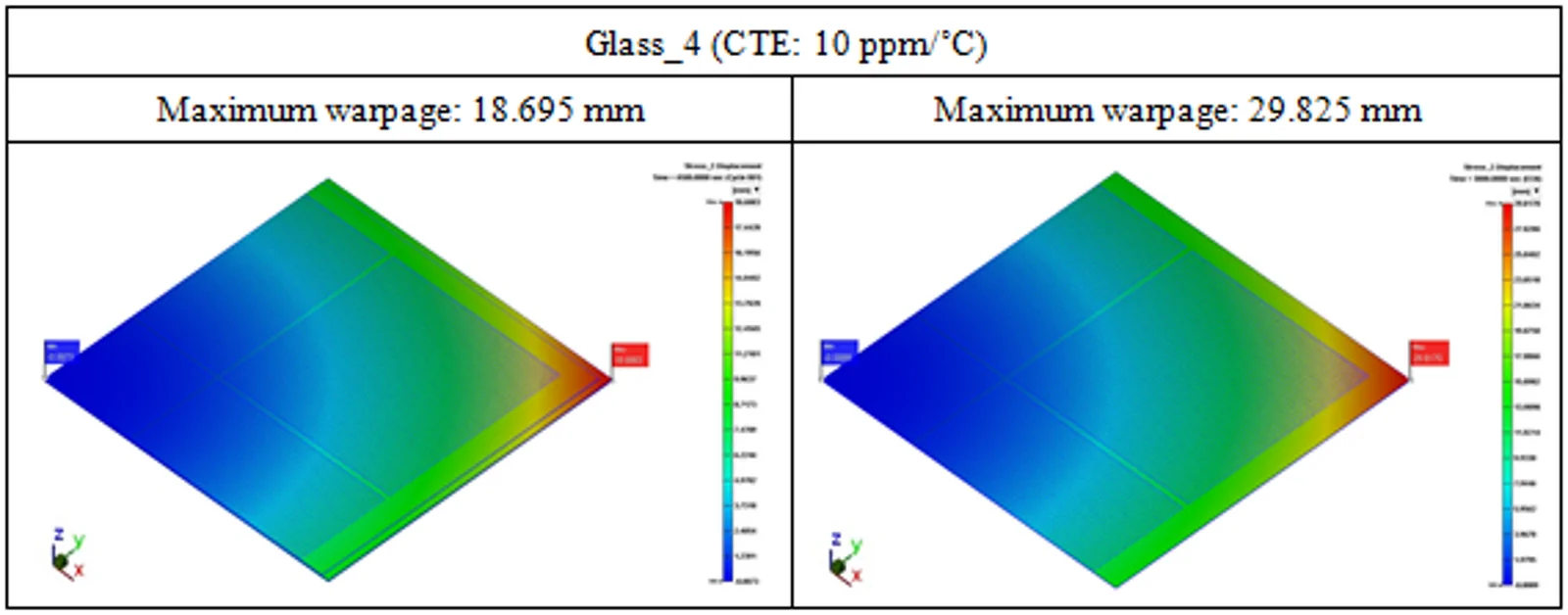
Comparison of molding-first warpage simulation results with different carriers.

**Table 10:** Warpage results comparison with different carrier material.

Carrier material	Coefficient of thermal expansion (ppm/°C)	Maximum warpage (mm)	
		Molding	Debonding
Ceramic	7.8	+ 1.158	+ 9.339
Steel_1	7.8	+ 2.787	+ 10.676
Steel_2	8.5	+ 1.815	+ 10.287
Steel_3	10	− 0.858	+ 9.453
Steel_4	12	− 2.366	+ 15.761
Glass_1	7	+ 27.727	+ 30.238
Glass_2	8	+ 24.717	+ 30.101
Glass_3	9.1	+ 20.474	+ 29.949
Glass_4	10	+ 18.695	+ 29.825

## Conclusions

This study developed an integrated finite element simulation framework to systematically investigate warpage behavior in fan-out panel-level packaging (FOPLP) under both RDL-first and molding-first process flows. The framework applied the element birth and death technique to replicate sequential material deposition and removal. By introducing equivalent and average reference temperature methodologies, the model accurately captured process-induced deformation from high-temperature curing to carrier debonding, achieving strong agreement with experimental measurements.

For the RDL-first process, the results demonstrate that process temperature plays a dominant role in determining the accuracy of warpage prediction. Simulations based on the actual process temperature progressively overestimate deformation as the number of layers increases, whereas the use of equivalent reference temperatures leads to systematic underestimation. The proposed average reference temperature effectively balances these discrepancies and reproduces the experimental trend, achieving an average error of 23.8%. Stress analysis further indicates that the maximum von Mises stress consistently localizes within the RM 1 and WAL layers, which correspond to commonly observed failure locations in practical redistribution layer structures. Despite the effectiveness of the proposed framework, certain limitations remain. The equivalent material approach introduces simplifications that may not fully capture localized microstructural effects, and the experimental validation is limited to specific process conditions.

For the molding-first process, the results reveal the coupled effects of thermal mismatch, mechanical stiffness, and cure shrinkage on package warpage. During compression molding, the deformation magnitude was primarily governed by the curing volume shrinkage of EMC, whereas the carrier’s stiffness provided mechanical restraint. An inverse relationship was observed between warpage and CTE, indicating that warpage decreased as CTE increased during the molding stage. After carrier debonding, the release of residual stresses caused substantial warpage growth, particularly in metallic carriers such as steel. In contrast, the ceramic carrier exhibited minimal deformation at both stages, confirming its superior dimensional stability.

A comparative evaluation between both process flows highlights that optimizing process temperature conditions, carrier CTE, and Young’s modulus is essential to control overall package deformation. A carrier with moderate CTE and high stiffness, such as ceramics, can effectively suppress warpage without introducing excessive residual stress, thus improving process yield and reliability. The modeling strategy presented in this work establishes a physics-based guideline for material selection and process design in large-panel packaging and can be extended to predict reliability behavior in future multi-die, high-density integration architectures.

## Data Availability

The datasets used and analyzed during the current study are available from the corresponding author on reasonable request.

## References

[1] Tanja Braun, K. F. et al. Challenges and opportunities for fan-out panel level packing (FOPLP). In *9th International Microsystems, Packaging, Assembly and Circuits Technology Conference (IMPAC)T*, 154–157 (2014).

[2] Stoney, G. G. The tension of metallic films deposited by electrolysis. In *Proceedings of the Royal Society of London. Series A, Containing Papers of a Mathematical and Physical Character*, vol. 82, no. 553, 172–175 (1909).

[3] Timoshenko, S. Analysis of bi-metal thermostats. *J. Opt. Soc. Am.* **11** (3), 233–255 (1925).

[4] Suhir, E. Predicted bow of plastic packages of integrated circuit (IC) devices. *J. Reinf. Plast. Compos.* **12** (9), 951–972 (1993).

[5] Schapery, R. A. Thermal expansion coefficients of composite materials based on energy principles. *J. Compos. Mater.* **2** (3), 380–404 (1968).

[6] Che, F. X., Ho, D., Ding, M. Z. & MinWoo, D. R. Study on process induced wafer level warpage of fan-out wafer level packaging. In *IEEE 66th Electronic Components and Technology Conference (ECTC)*, 1879–1885 (2016).

[7] Chen, Y. H., Lin, P. B., Ko, C. T. & Tseng, T. J. Innovated ultra-thin and super fine-pitch panel RDL substrate manufacturing for advanced package. In *International Conference on Electronics Packaging (ICEP)*, 16–18 (2017).

[8] Wu, M.-L. & Lan, J. S. Simulation and experimental study of the warpage of fan-out wafer-level packaging: The effect of the manufacturing process and optimal design. *IEEE Trans. Compon. Packag. Manuf. Technol.* **9** (7), 1396–1405 (2018).

[9] Lee, C. C., Wang, C. W. & Chen, C. Y. Comparison of mechanical modeling to warpage estimation of rdl-first fan-out panel-level packaging. *IEEE Trans. Compon. Packag. Manuf. Technol.* **12** (7), 1100–1108 (2022).

[10] Liang, C. W. et al. Fan-out panel-level package warpage and reliability analyses considering the fabrication process. *J. Manuf. Process.* **119**, 649–665 (2024).

[11] Lee, M. K., Jeoung, J. W., Ock, J. Y. & Choa, S. H. Numerical analysis of warpage and reliability of fan-out wafer level package. *J. Microelectron. Packag. Soc.* **21** (1), 31–39 (2014).

[12] Tsai, C. H., Liu, S. W. & Chiang, K. N. Warpage analysis of fan-out panel-level packaging using equivalent CTE. *IEEE Trans. Device Mater. Reliab.* **20** (1), 51–57 (2019).

[13] Teng, S.-Y. & Hwang, S. J. Simulations of process-induced warpage during IC encapsulation process. *J. Electron. Packag.* 307–315(2007).

[14] Deng, S. S., Hwang, S. J. & Lee, H. H. Warpage prediction and experiments of fan-out wafer level package during encapsulation process. *IEEE Trans. Compon. Packag. Manuf. Technol.* **3** (3), 452–458 (2013).

[15] Chen, H. L. & Chiang, K. N. The effect of geometric and material uncertainty on debonding warpage in fan-out panel level packaging. In *24th International Conference on Thermal, Mechanical and Multi-Physics Simulation and Experiments in Microelectronics and Microsystems*, 2023 (2023).

[16] Lo, C. H., Chang, T. Y., Lee, T. Y. & Hwang, S. J. Analysis of warpage and reliability of very thin profile fine pitch ball grid array. *Heliyon*. **10**, 15 (2024). 10.1016/j.heliyon.2024.e35459 PMC11334874 39166078

[17] Lee, T. Y., Chen, Y. L., Hwang, S. J., Ko, C. Y. & Cheng, W. L. Viscoelastic warpage and stress analysis in SIP packaging: Numerical simulation and experimental validation. *Results Eng.* **26**, (2025).

[18] Khosravani, M., Reza & Reinicke, T. Experimental characterization of 3D-printed sound absorber. *Eur. J. Mech.-A/Solids*. **89**, (2021).

[19] Khosravani, M. et al. Brittle fracture of notched components fabricated by stereolithography. *J. Mater. Res. Technol.* **33**, 8071–8078 (2024).

[20] Khosravani, M., Reza, P., Soltani & Reinicke, T. Effects of steps on the load bearing capacity of 3D-printed single lap joints. *J. Mater. Res. Technol.* **23**, 1834–1847 (2023).

[21] Yahui Lyu, A. et al. Enhanced mechanical performance of 3D printed continuous carbon fibre reinforced polyphenylene sulphide composites through dopamine treatment and post-processing compression. *Compos. Part. A Appl. Sci. Manuf.* **190**, (2025).

[22] Castro, J. & Macosko, C. Kinetics and rheology of typical polyurethane reaction injection molding systems. In *Proceedings of the 38th Society of Plastics Engineers Technical Papers Annual Technical Conference, Plastics Progress Through Process* (1980).

[23] Kamal, M. R. & Ryan, M. The behavior of thermosetting compounds in injection molding cavities. *Polym. Eng. Sci.* **20** (13), 859–867 (1980).

[24] Chang, Y. S., Hwang, S. J., Lee, H. H. & Huang, D. Y. Study of PVTC relation of EMC. *J. Electron. Packag.* **124** (4), 371–373 (2002).

[25] Hwang, S. J. & Chang, Y. S. Isobaric cure shrinkage behaviors of epoxy molding compound in isothermal state. *J. Polym. Sci. Part B Polym. Phys.* **43** (17), 2392–2398 (2005).

